# Periodontal disease is associated with the risk of cardiovascular disease independent of sex: A meta-analysis

**DOI:** 10.3389/fcvm.2023.1114927

**Published:** 2023-02-27

**Authors:** Yurong Leng, Qinwen Hu, Qin Ling, Xiongda Yao, Menglu Liu, Jiawei Chen, Zhiwei Yan, Qun Dai

**Affiliations:** ^1^The Affiliated Stomatological Hospital of Nanchang University, Nanchang, Jiangxi, China; ^2^The Key Laboratory of Oral Biomedicine, Nanchang, Jiangxi, China; ^3^Jiangxi Province Clinical Research Center for Oral Disease, Nanchang, Jiangxi, China; ^4^The Second Clinical Medical College of Nanchang University, Nanchang, Jiangxi, China; ^5^Department of Cardiology, Seventh People’s Hospital of Zhengzhou, Zhengzhou, China; ^6^Department of Sports Rehabilitation, College of Human Kinesiology, Shenyang Sport University, Shenyang, China

**Keywords:** periodontal disease, cardiovascular disease, epidemiology, sex difference, prevalence

## Abstract

**Objectives:**

Studies have established a link between periodontal disease and cardiovascular disease (CVD), but it is unclear whether there is a sex difference in their association.

**Methods:**

The PubMed, Embase, and Cochrane databases were searched until June, 21 2022. Cardiovascular outcomes included any CVD, myocardial infarction (MI), coronary heart disease (CHD), or stroke. Studies reported the prevalence of CVD in patients with periodontal disease and the relationship between periodontal disease and CVD. The study is registered with PROSPERO (CRD42022333663). The level of evidence and recommendations is assessed by the Grading of Recommendations for Assessment, Development and Evaluation (GRADE).

**Results:**

Twenty-six studies were included. In patients with periodontal disease, the prevalence of CVD was 7.2% [9 studies; 95% confidence interval (CI): 2.7–13.6%], and prevalence for CHD, hypertension, stroke, and heart failure was 6.6, 25.3, 1, and 1.1%, respectively. There was a significant association between periodontal disease and CVD in men [odds ratio (OR) = 1.22; 95% CI: 1.12–1.34] and women (OR = 1.11; 95% CI: 1.05–1.17), with no significant sex difference (*P* > 0.05).

**Conclusion:**

Cardiovascular disease is relatively common in patients with periodontal disease, and an increased risk of CVD is associated with periodontal disease independent of sex. Interventions targeting periodontal disease may be beneficial for CVD.

**Systematic review registration:**

https://www.crd.york.ac.uk/PROSPERO/, identifier CRD42022333663.

## Introduction

Cardiovascular disease (CVD) remains the leading cause of death, accounting for approximately one third of all deaths worldwide. The global incidence of CVDs is 10∼30%, showing a gradually increasing trend (1–3). China has the highest cardiovascular mortality rate, followed by India, the Russian Federation and the United States of America (4). However, periodontal disease is increasingly becoming one of the major obstacles to optimal outcomes for patients with cardiovascular disease (CVD) (5).

Periodontal disease is one of the most common inflammatory diseases in humans that destroys hard and soft tissues around the tooth, resulting in tooth loosening and loss (6). Severe periodontal disease affects 10.8% of the world’s population and is the sixth most common disease worldwide, with more than 700 million people suffering from severe periodontal disease (7). Periodontal disease can cause inflammation of periodontal tissue but also produce inflammatory mediators and can products that can cardiovascular health through blood circulation (8). In recent decades, longitudinal studies have revealed a firm link between periodontal tissue and an increased risk of CVD. However, no study has systematically studied the prevalence of CVD in patients with periodontal disease. Moreover, female sex is considered to have a protective effect on the incidence and development of CVD (9, 10). However, whether there is a sex difference in the relationship between periodontal disease and the risk of CVD remains unexplored. Hence, the present study aimed to (i) systematically evaluate the prevalence of CVD in patients with periodontal disease, and (ii) examine the sex-specific association of periodontal disease with CVD.

## Materials and methods

### Protocol registration

The study has been registered with PROSPERO (International Registry of Prospective Systems Review: https://www.crd.york.ac.uk/PROSPERO/ number: CRD42022333663). This meta-analysis was conducted in accordance with PRISMA 2021 guidelines for systems evaluation and meta-analysis 1 (11) (Supplementary Table 1).

### Search strategy

The PubMed, Embase, and Cochrane Library online databases were searched up to June 25, 2022 with no language restriction. The MeSH search items and keywords were as follows: [“periodontal disease” (MeSH) OR “furcation defects” OR “gingival diseases” OR “peri-implantitis” OR “periapical diseases” OR “periodontal atrophy” OR “periodontal cyst” OR “periodontitis” OR “tooth migration” OR “periodontitis” OR “tooth mobility” OR “tooth loss”] AND [“cardiovascular diseases” (MeSH)]. The detailed search strategy is shown in Supplementary Table 2.

### Study selection

After a database search, the retrieved studies were imported into Endnote X9 software (Thomson Reuters, New York, NY, USA). The title and abstract were selected by one author (YL) and verified by a second author (QH). Before data extraction and quality assessment, the whole article was qualified. The authors reached a consensus on the included studies, and the differences were resolved through in-depth discussion. Before the database search, a data extraction form was developed to identify key study information, including demographics, data sources, exclusion criteria, follow-up periods, diagnostic criteria, and outcome measures.

Strict eligibility criteria guided the search to ensure relevant study inclusion, reduce heterogeneity and increase the power of the results. The inclusion criteria for epidemiological studies were as follows: (1) adult’s patients with periodontal diseases (including periodontitis and gingivitis); (2) reported prevalence of CVD in patients with periodontal diseases; and (3) cross-sectional, retrospective, or prospective cohort and randomized controlled trials studies. Studies with sample size <10,000 were excluded.

Additionally, eligible criteria for studies of the relationship between periodontal disease and CVD were as follows according to the PICOS: (1) Types of participants: adults; (2) Exposure and comparator: patients with periodontal disease vs. without periodontal disease; and (3) Outcomes: sex-specific association between periodontal diseases and CVD. (4) Types of studies: retrospective or prospective cohort, case-control and randomized controlled trials studies. Studies were excluded from the review if they met any one of the following criteria: (1) Protocols, reviews, conference abstracts, or animal studies; (2) Studies with unavailable data even after contacting the corresponding author for further information.

### Data extraction and quality assessment

Two authors (YL and QH) extracted relevant information from each study: (1) first author; (2) publication year; (3) country; (4) study design; (5) follow-up period; (6) basic characteristics of the population (sample size, mean age); (7) diagnosis for periodontal diseases; (8) outcomes; (9) adjustments; and (10) RR or OR with 95% CI in the adjusted model.

The Newcastle-Ottawa quality scale (NOS) was used to quantify the quality of cohort studies, with a score above six regarded as acceptable quality. We evaluated the quality and strength of the evidence using the Grading of Recommendations Assessment, Development and Evaluation (GRADE) method, which evaluates each outcome according to the recommended rating assessment, development and evaluation. Two authors (QH and YL) evaluated the quality of evidence of each result, providing an evidence profile table by GRADE profiler software. The results were described in the outcome measure type section, whose footnotes were used to justify any decision to reduce or improve the quality of the evidence.

### Statistical analysis

We treated RRs and HRs as equivalent to ORs, and pooled the summary ORs with corresponding 95% CIs using the inverse-variance method (12). Random effect meta-analysis was performed for the overall as well as separate CVD outcomes.

Heterogeneity was evaluated using the Higgins I-squared (I^2^) statistic (30, 50, and 75% represent low, moderate, and high heterogeneity, respectively) (13). Publication bias was addressed by the funnel plot, and Egger’s and Begg’s tests. To appraise the robustness and reliability of the primary study outcomes, we also carried out sensitivity analyses by omitting each study in turn. The statistical analysis was performed by RevMan software, version 5.4.1 (The Cochrane Collaboration, Nordic Cochrane Center, Copenhagen, Denmark) and Stata software, Version 16.0 (Stata Corp. LP, College Station, TX, USA). *P* < 0.05 double-sided was considered statistically significant.

## Results

### Literature search

**FIGURE 1:**
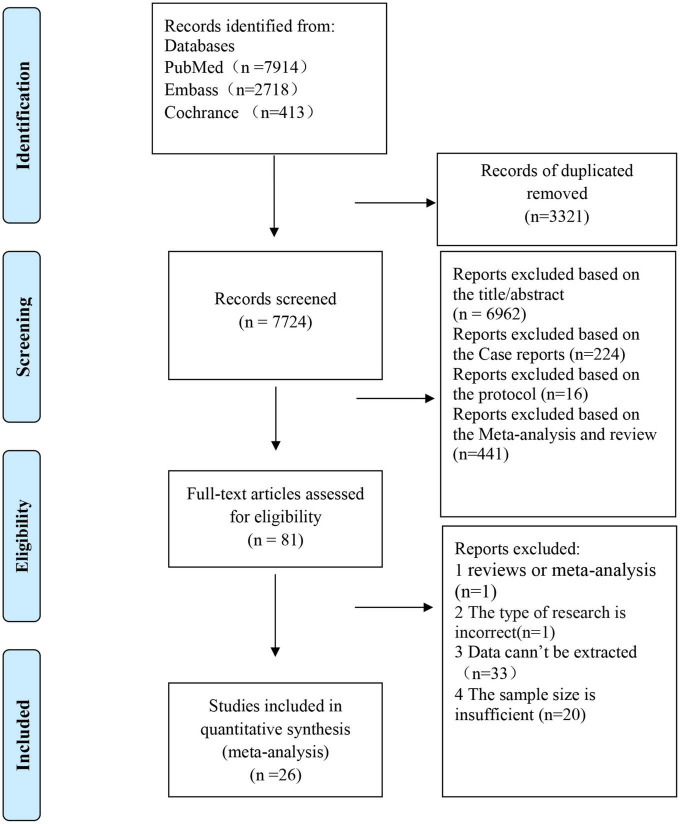
Flowchart of the study selection for the meta-analysis of an association between periodontal disease and cardiovascular disease [adapted from Moher et al. (67)].

As shown in Figure 1, an initial online database search resulted in 11,045 articles. After excluding 3,321 duplicated records and 7,643 irrelevant studies, 81 studies remained for full-text review. Fifty-five studies are excluded for the following reasons: (1) reviews or meta-analysis (*n* = 1); (2) without target data (*n* = 33); (3) without sufficient sample size (*n* = 20); (4) The type of research is inappropriate (*n* = 1). The excluded studies with detailed reasons were listed in Supplementary Table 3. Finally, 26 (14–39) studies were included in this meta-analysis, of which nine (14–22) were epidemiological studies and 20 (16, 17, 22–39) reported a sex-specific association between periodontal disease and CVD.

### Study characteristics and quality

Table 1 summarizes the main characteristics of the included studies. The included studies were published between 1993 and 2019, and the sample sizes ranged from 1,231 to 626,106. Most studies were conducted in North America (*n* = 15), with seven and three being from Asia and Europe, respectively, while the remaining study was from Europe. Fifteen were prospective cohort studies, five were case-control studies and six were retrospective cohort studies. The follow-up time ranged from 2 years to 21 years. Sixteen (14, 16, 18–21, 23–27, 29, 33, 39–41) studies defined periodontal disease according to the Oral Hygiene Index-Simplified (OHI-S), and the remaining 10 (17, 22, 30–32, 34–38) studies were identified based on self-report. All studies defined CVD according to the International Classification of Diseases. Adjustments varied widely among the studies reporting a sex-specific association between periodontal disease and CVD, while the primary confounding factors were age, sex, smoking, alcohol consumption, hypertension (HP), diabetes, and heart failure (HF). Based on the NOS, twenty studies (16, 17, 22–39) reporting the association of periodontal disease and CVD were considered to be of moderate to high quality, with a score range of 6–9, and one (29) study had a score of 5 (Supplementary Table 4).

**TABLE 1:** Characteristics of included studies in this meta-analysis.

References, Country	Study design	Total population	Case (M/F)	Age (years)	Exposure/PD diagnosis	Outcome	Total study follow-up period (years)	OR/RR (95% CI)	Outcomes included	Adjusting factors
Yu et al. (22), USA	Prospective cohort	39,863	14,370 F	≥45	PD/self-report	MI	16	1.65 (1.11–2.45)	Relationship between PD and CVD	Age, race, BMI, education, smoking, diabetes, HP, hypercholesterolemia, family history of MI, PA
						Stroke		1.28 (0.87–1.88)		
						Stroke + MI (Total CVD)		1.2 (0.97–1.49)		Major CVD, MI, ischemic stroke, and total CVD
Batty et al. (24), Korea	Prospective cohort	349,579	52,825 F	45.7	PD/clinical	CHD	21	1.03 (0.98–1.08)	Relationship between PD and CVD	Alcohol intake, smoking, exercise, SBP, fasting blood cholesterol, diabetes, BMI, family history of CVD
		626,106	187,004 M	45.4				1.03 (1.01–1.05)		
Beck et al. (25), USA	Case control	1,147	486 M	45.7	PD/clinical	Fatal CHD	18	1.9 (1.10–3.43)	Relationship between PD and CVD	Age, smoking, NIDDM, DBP, family history, education
						Stroke		2.8 (1.45–5.48)		
						CHD + Stroke		1.69 (1.26–2.26)		
Choe et al. (26), Korea	Prospective cohort	679,170	41,859 F	42.6	PD/clinical	Stroke	14	1.1 (1.09–1.11)	Relationship between PD and CVD	Age, obesity, hypercholesterolemia, HP, diabetes, alcohol drinking, exercise, and smoking
DeStefano et al. (27), USA	Prospective cohort	9,760	1,486 M	25–49	PD/clinical	Total mortality	16	1.46 (1.26–1.7)	Relationship between PD and CVD	Age, sex, race, education, poverty index, marital state, SBP, TC, diabetes, BMI, PA, alcohol, and smoking
						CHD		1.25 (1.06–1.48)		
Dietrich et al. (28), USA	Prospective cohort	1,203	309 M	50	PD/clinical	CHD	24	1.31 (0.96–1.78)	Relationship between PD and CVD	Age, BMI, HDL, TC, TG, HP, mean SBP, DBP, DM, fasting glucose, smoking, alcohol, occupation, education, income, and marital status
Heitmann and Gamborg (29), Denmark	Prospective cohort	2,932	632 M	35–65	PD/clinical	CHD	7	1.4 (1.09–1.81)	Relationship between PD and CVD	Education, age, smoking, diabetes, alcohol intake, SBP, and BMI
			622 F			CHD		1.25 (0.93–1.68)		
Howell et al. (30), USA	Retrospective cohort	22,037	5,306 M	40–84	PD/clinical	MI	13	1.01 (0.8–1.28)	Relationship between PD and CVD	Age, aspirin and beta-carotene treatment assignment, smoking, alcohol, HP, history of treatment for HP, MI, angina, BMI, diabetes, PA
						Stroke		1.01 (0.81–1.27)		
						Stroke + MI		1.01 (0.88–1.15)		
Hung et al. (32), USA	Retrospective cohort	51,529	7,313 M	40–75	PD/clinical	PAD	12	1.19 (0.70–2.03)	Relationship between PD and CVD	Age, smoking, alcohol, BMI, PA, MI, multivitamin supplement, vitamin E, HP, diabetes, hypercholesterolemia
Hung et al. (31), USA	Prospective cohort	41,407	6,619 M	40–75	PD/clinical	CHD	12	1.46 (1.03–2.09)	Relationship between PD and CVD	Age, smoking, alcohol, BMI, PA, MI, multivitamin supplement use, vitamin E, history of women only, menopausal, HP, diabetes, hypercholesterolemia, professions for men only, for women only, menopausal, and hormone use.
Jimenez et al. (33), USA	Prospective cohort	1,231	720 M	27–84	PD/self-report	Stroke	34	1.07 (0.9–1.27)	Relationship between PD and CVD	Age, BMI, HDL, TC, TG, HP, SBP, DBP, diabetes, alcohol, smoking, marital status, and baseline measures of education, occupation, and income
Joshipura et al. (34), USA	Prospective cohort	41,380	6,613 M	40–75	PD/clinical	Stroke	12	1.55 (1.27–1.99)	Relationship between PD and CVD	Age, smoking, alcohol, BMI, PA, MI, multivitamin supplement, vitamin E, HP, diabetes, hypercholesterolemia, and professions updated foreach 2-year time period
Joshipura et al. (35), USA	Prospective cohort	44,119	7,040 M	40–79	PD/clinical	CHD	6	1.01 (0.94–1.29)	Relationship between PD and CVD	misclassification in the self-reported measure and prevalence
LaMonte et al. (17), USA	Prospective cohort	57,001	14,847 F	55–89	PD/self-report	CHD	12	1.08 (0.97–1.20)	Relationship between PD and CVD	Age, smoking, dental visits, diabetes, race, education, HP, HC, BMI, PA, alcohol, dietary healthy eating index, and CVD
						Stroke		1.11 (0.95–1.30)		
						CHD + Stroke		1.06 (0.98–1.13)		
Noguchi et al. (36), Japan	Case control	3,081	739 M	36–59	PD/self-report	MI	5	2.26 (0.84–6.02)	Relationship between PD and CVD	Age, BMI, smoking, HP, diabetes, dyslipidemia, and family history of heart disease
Rivas-Tumanyan et al. (37), USA	Prospective cohort	31,543	4,641 M	40–75	PD/self-report	HP	20	1.07 (1.01–1.13)	Relationship between PD and CVD	Age, smoking, HP, race, dental profession, diabetes, alcohol, BMI, PA, fruit and vegetable intake, vitamin E, vitamin D, and calcium intake, and multivitamin supplement use
Senba et al. (38), Japan	Case control	29,904	6,816 M	<45	PD/self-report	MI	18	2.34 (1.05–5.23)	Relationship between PD and CVD	With and without CHD
			23,088 F			MI		1.76 (0.64–4.28)		
			6,816 M			CHD		1.69 (1.02–2.81)		
			23,088 F			CHD		1.62 (1.04–2.53)		
			6,816 M			AP		1.17 (0.57–2.43)		
			23,088 F			AP		1.75 (1.04–2.95)		
Andriankaja et al. (23), USA	Case control	1,461	415 M	35–69	PD/clinical	MI	5	1.34 (1.15–1.57)	Relationship between PD and CVD	Age, gender, HP, cholesterol, diabetes, BMI, PA, smoking
			120 F				5	2.08 (1.47–2.94)		
Tuominen et al. (39), Finland	Case control	4,910	2,518 M	30–69	PD/clinical	CHD death	12	1.00 (0.6–1.6)	Relationship between PD and CVD	Age, other oral health and indicators, education, HP, hypercholesterolemia, smoking, and diabetes
			2,392 F			CHD death	12	0.9 (0.20–2.1)		
Lee et al. (16), Taiwan	Retrospective cohort	719,436	247,515 M	≥20	PD/clinical	Stroke	11	1.33 (1.29–1.37)	Relationship between PD and CVD	Age
			263,247 F					1		
Morrison et al. (14), Canada	Prospective cohort	14,534	23	35–69	PD/clinical attachment loss	CHD death	21	3.3 (1.11–10.4)	Prevalence of CVD	Age, sex, TC, smoking, diabetes, hypertensive status, and province of residence
		4,559	40	70–84				0.86 (0.46–1.61)		
Beck et al. (15), USA	Prospective cohort	15,792	NA	45–64	PD/clinical	HP	NA	1.9 (1.50–2.33)	Prevalence of CVD	Age, sex, race, diabetes, HP, waist-to-hip ratio, HDL, LDL, TG, education, smoking
Chen et al. (20), Taiwan	Retrospective cohort	393,745	8,138	≥65	PD/clinical	AF	2	1.10 (1.06–1.14)	Prevalence of CVD	Age, gender, outpatient visits, dental scaling frequency, and comorbidities
Hansen et al. (19), Danish	Retrospective cohort	5,536,422	NA	57.3	PD/clinical	MI	15	1.16 (1.04–1.30)	Prevalence of CVD	Age, sex, smoking, comorbidities, medication, and socio-economic
						CVD		2.02 (1.87–2.18)		
						Stroke		1.51 (1.38–1.65)		
						All CVD		2.7 (2.60–2.81)		
Joshy et al. (18), Australian	Prospective cohort	9,802	23	≥45	PD/clinical	Stroke	5	1.2 (0.9–1.62)	Prevalence of CVD	Age, sex, smoking, alcohol, Australian born status, region of residence, education, health insurance, PA, and BMI
LaMonte et al. (17), USA	Prospective cohort	14,847	949	55–89	PD/self-report	Total CVD	12	1.06 (0.98–1.13)	Prevalence of CVD	Age, smoking dental visits, diabetes, race, education, HP, HC, BMI, PA, alcohol, dietary healthy eating index. CVD indicates cardiovascular disease
			450			CHD		1.08 (0.97–1.20)		
			226			Stroke		1.11 (0.95–1.30)		
			1,090			Total mortality		1.12 (1.05–1.21)		
			213			CVD mortality		1.09 (0.93–1.28)		
Lee et al. (16), Taiwan	Retrospective cohort	45,296	60	20–44	PD/clinical	Stroke	11	2.17(1.64–2.87)	Prevalence of CVD	Treatment and without treatment
			278	45–64				1.19(1.05–1.35)		
			476	≥65				1.13(1.03–1.25)		
Ahn et al. (21), Korea	Retrospective cohort	14,625	139	19–29	PD/clinical	HT	NA	1	Prevalence of CVD	Age, sex, drinking, smoking, and PA
			618	30–39				2.84 (2.38–3.38)		
			1,134	40–49				5.22 (4.42–6.16)		
			1,266	50–59				6.72 (5.69–7.93)		
			2,016	≥60				6.53 (5.52–7.72)		

CHD, coronary heart disease; CVD, cardiovascular disease; PAD, peripheral arterial disease; HP, hypertension; AP, angina pectoris; MI, myocardial infarction; AF, atrial fibrillation; BMI, body mass index; TC, total cholesterol; HC, high cholesterol; TG, triglycerides; LDL, low density lipoprotein; HDL, high-density lipoprotein; RSBP, resting systolic blood pressure; SBP, systolic blood pressure; DBP, diastolic blood pressure; PA, physical activity; NIDDM, non-insulin-dependent diabetes mellitus; NA, not application.

### Epidemiology of CVD in patients with periodontal disease

**FIGURE 2:**
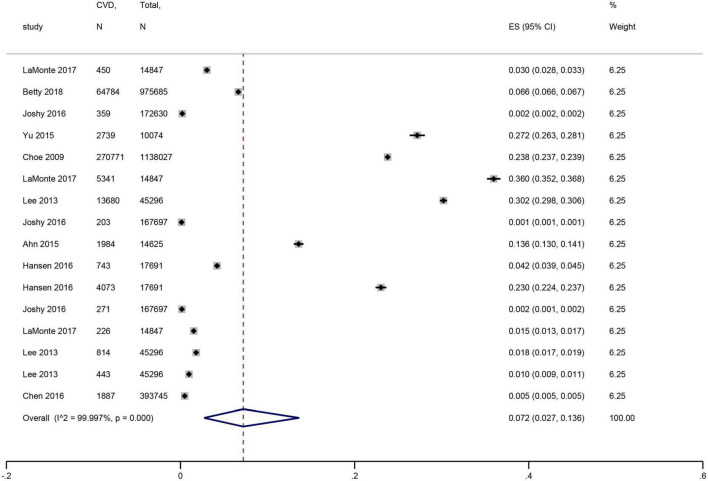
Forest plot of the prevalence of cardiovascular disease in patients with periodontal disease. In the forest plot, the diamond indicates the pooled estimate. Red boxes are relative to study size and the black vertical lines indicate 95% CIs around the effect size estimate.

Nine studies (15–21, 24, 26) with 2,737,324 participants were included. The pooled prevalence of CVD in periodontal disease was 7.2% (95% CI: 2.7–13.6%), with high heterogeneity (*I*^2^ = 99.997%) (Figure 2).

**FIGURE 3:**
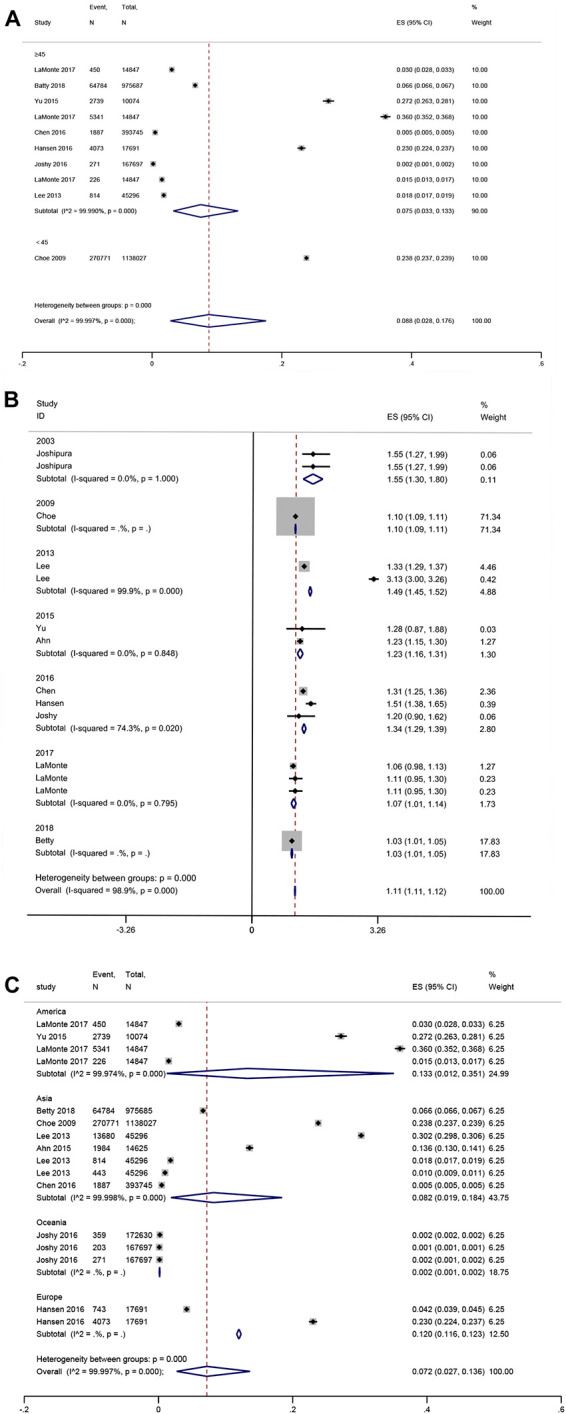
Forest plot of subgroup analysis of prevalence of cardiovascular disease in patients with periodontal disease [**(A)** Age, **(B)** Publication years, and **(C)** Region]. In the forest plot, the diamond indicates the pooled estimate. Red boxes are relative to study size and the black vertical lines indicate 95% CIs around the effect size estimate.

In the subgroup analysis, younger patients (mean age <45 years) with periodontal disease showed a higher prevalence of CVD than older patients (mean age ≥45 years), whose effect size (ES) was 23.8 vs. 7.5% (*P* for subgroup difference < 0.001) (Figure 3A). Furthermore, no significant difference was shown according to the publication years (*P* = 1) (Figure 3B). Americans had the highest prevalence of CVD (ES: 13.3%), followed by Europeans (ES: 12%), Asians (ES: 8.2%), and Oceania (ES: 2%) (*P* for subgroup difference < 0.001) (Figure 3C). The pooled prevalence was 25.3% for HP, 6.6% for coronary heart disease (CHD), 1% for stroke, and 1.1% for HF (Supplementary Figure 1).

### Sex-specific association between CVD and periodontal disease

**FIGURE 4:**
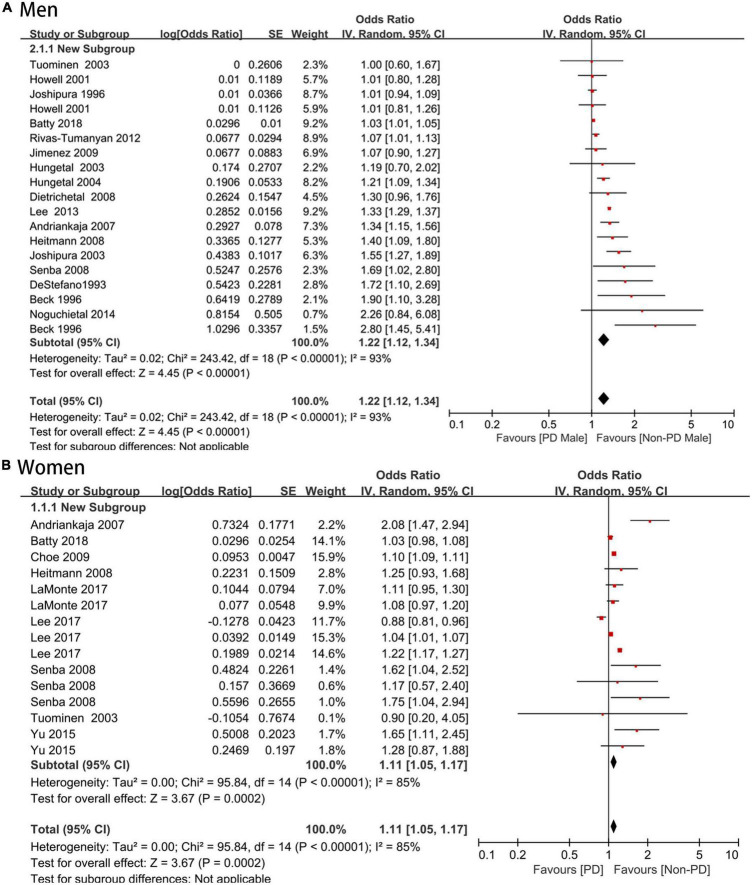
**(A,B)** Forest plot of the risk of cardiovascular disease in periodontal disease patients by sexes. In the forest plot, the diamond indicates the pooled estimate. Red boxes are relative to study size and the black vertical lines indicate 95% CIs around the effect size estimate.

Seventeen (16, 23–25, 27–39) studies with 1,308,625 men reported an association between periodontal disease and CVD (OR = 1.22; 95% CI: 1.12–1.34), with high heterogeneity (*I*^2^ = 93%; *P* < 0.001) (Figure 4A). Nine (17, 22–24, 26, 29, 38, 39) studies with 1,990,952 women reported an association between periodontal disease and CVD (OR = 1.11; 95% CI: 1.05–1.17; *I*^2^ = 85%; *P* = 0.0002) (Figure 4B), and there was no significant sex difference (*P* for interaction > 0.05).

### Sex-specific association between periodontal disease and coronary artery disease

**FIGURE 5:**
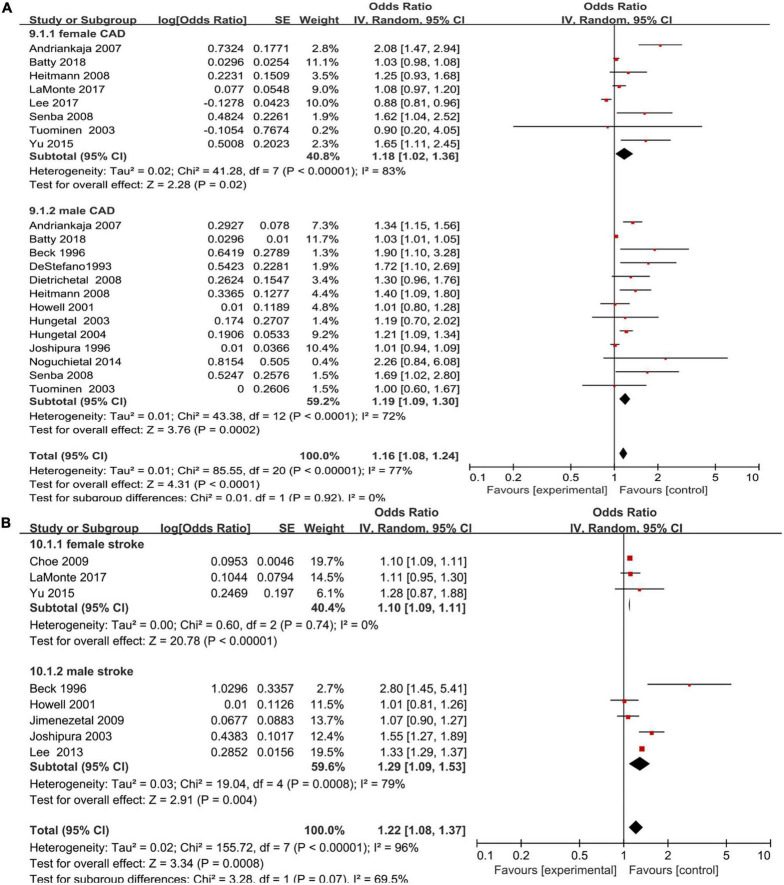
Forest plot of the risk of coronary artery disease and stroke in periodontal disease patients by sex [**(A)** Coronary artery disease and **(B)** stroke]. In the forest plot, the diamond indicates the pooled estimate. Red boxes are relative to study size and the black vertical lines indicate 95% CIs around the effect size estimate.

An increased risk of coronary artery disease was found in male periodontal disease patients (OR = 1.19; 95% CI: 1.09–1.3) with moderate heterogeneity (*I*^2^ = 72%), which was consistent with the female group (OR = 1.18; 95% CI: 1.02–1.36; *I*^2^ = 83%), and no significant sex difference was shown (*P* > 0.05) (Figure 5A).

### Sex-specific association between periodontal disease and stroke

For eight studies that reported an association between periodontal disease and stroke, there was a higher risk of stroke (16, 17, 22, 25, 26, 30, 33, 34) in both male and female periodontal disease patients (OR = 1.29, 95% CI: 1.09–1.53; *I*^2^ = 79% versus OR = 1.10, 95% CI: 1.09–1.11, *I*^2^ = 0%) with no significant sex difference (*P* > 0.05) (Figure 5B).

### Publication bias and sensitivity analysis

As shown in Supplementary Figure 2, although some presence of bias was found by funnel plots, Egger’s test (*P* > 0.1), and Begg’s test (*P* > 0.1) showed no significant publication bias. Sensitivity analysis by omitting each study showed that our results were stable and reliable, with a range from 1.09 (95% CI: 1.03–1.15) to 1.14 (95% CI: 1.05–1.24) for the relationship between periodontal disease and CVD in females, and 1.24 (95% CI: 1.12–1.38) to 1.29 (95% CI: 1.15–1.43) for the relationship between periodontal disease and CVD in males (Supplementary Figure 3).

### Quality assessment

The GRADE tool was used to evaluate the quality of evidence. All the included studies were observational studies; thus, so the initial level of evidence was low (42). Ultimately, the risk of CVD was evaluated as very low certainty (Supplementary Table 5).

## Discussion

### Major findings

The present meta-analysis showed that the pooled prevalence was 7.3% for CVD, 6.6% for CHD, 25.3% for HP, 1% for stroke, and 1.1% for HF among periodontal disease patients. Moreover, a significant association between periodontal disease and the risk of CVD was found independent of sex, with a summary OR of 1.22 (95% CI: 1.12–1.34) for females and 1.11 (95% CI: 1.05–1.17) for males. To the best of our knowledge, this article is the first to explore the sex-specific association of periodontal diseases and CVD.

Consistent with results of a previous meta-analysis (43), our study also showed that periodontal disease is associated with the risk of CVD. More importantly, our meta-analysis also showed that this association remains in both men and women, without sex differences. To ensure the rigor of our results, necessary analysis for potential publication bias was conducted, and the final results were reliable. CVD has always been shown to have a greater possibility of secondary oral problems. As Lazureanu et al. showed in their research on the prevalence of periodontal disease in patients with CVD (44), 77.5% of the 147 patients with CVD developed periodontal disease, implying a high incidence of periodontal disease among patients with CVD. However, few related studies have explored the incidence of CVD in patients with periodontal disease. Our study indicated that the prevalence of CVD in patients with periodontal disease reached 7.2%, suggesting that the occurrence of CVD is relatively common in patients with periodontal disease. As a result, we hypothesize that there may be a significant association between periodontal disease and CVD, which was also confirmed in our subsequent analysis about the association between periodontal disease and CVD.

Age is a vital risk factor for both CVD and periodontal disease. In subgroup analyses, younger patients with periodontal disease were shown to have an increased risk of CVD, with a prevalence of 23.8% in the younger group (age <45 years) versus 7.5% in older one (age ≥45 years). This was consistent with Joshipura et al. (34) who reported a stronger effect for younger men than for older men (≤55 years: RR = 2.17; 95% CI: 1.22–3.84 vs. >55 years: RR = 1.21; 95% CI: 0.92–1.59). Similarly, in a case-control study, four cases of clinical attachment loss of 4.5 to ≤6 mm were associated with a significantly increased risk of stroke (OR = 3.43; 95% CI: 1.39–8.50) in men ≤60 years, while there was no statistical significance in younger men (age >60 years) (OR = 1.71; 95% CI: 0.65–4.5). Men who were susceptible to periodontitis were shown to develop periodontal destruction earlier than those who were not. Thus, there is a stronger association between periodontitis and cerebrovascular disease in young men than in older men, which may further support the hypothesis of proinflammatory susceptibility.

### Potential mechanism

Our results showed that periodontal disease is strongly associated with a high risk of CHD, MI, and CVD, suggesting that periodontal disease is significantly associated with CVD. Systemic inflammation may be the core mechanism to explain the association between periodontal disease and the increased risk of CVD. Considerable studies have shown that inflammation is a predisposing factor or cardiovascular disease (45, 46), and patients with periodontal disease have been confirmed to have a high CRP lever as well as other inflammatory markers in the circulation (47, 48), indicating that it may lead to systemic inflammation and thus induce the development of CVD. In addition, oral pathogens such as *Porphyromonas gingivalis* (*P. gingivalis*) can reach the blood by crossing the gingival epithelial-conjunctival barrier and vascular endothelial cells, thus aggravating the inflammation and immune response in the original atherosclerosis in blood vessels (49–52). In addition, one animal study had found that *P. gingivalis* can enhance the expression of high mobility group box 1 (HMGB1) in mice after myocardial infarction. HMGB1 is a nuclear protein that induces inflammation (53). Therefore, it can be inferred that the increased risk of myocardial infarction caused by periodontal disease may be related to HMGB-1. One study showed that *P. gingivalis* infection and invasion can accelerate programmed cell death and the role of myocardial matrix metalloproteinases 9, which is not conducive to the recovery process of myocardial infarction prognosis, and may eventually lead to cardiac rupture (54). The increased risk of stroke may be due to decreased vascular endothelial function in the gingival tissue after infection with periodontal disease. The damage of vascular function may be caused by the basic interaction between oxidative stress and nitric oxide induced by periodontal disease (55).

### The effect of sex

Although our results showed that there was no difference between periodontal disease and CVD in different sexes, sex differences were shown in the coronary artery disease subgroup analysis, where men with periodontal disease had a higher risk of coronary artery disease than in women. In addition, a prospective cohort showed that the incidence of stroke in men was significantly higher than that in women in the total population and among the periodontal disease groups (56). This may arise from the expression of Y-encoded genes and the lack of cardiovascular protective effects of estrogen, leading to male-specific cardiovascular events. However, studies have also showed that female periodontal disease patients may have a higher risk of several CVDs. For example, an observational study showed that women with periodontal disease were 108% more likely to develop CVD (OR = 2.08, 95% CI: 1.47–2.94), which was higher than that of male periodontal disease patients (OR = 1.34, 95% CI: 1.15–1.57) (23). As a result, the sex-specific association between periodontal disease and CVD may be different in various types of CVDs. The mechanisms of how sex affects the relationship between periodontal disease and CVD are complex and still unclear, thus, further analysis is needed in the future. However, close monitoring of CVD in patients with periodontal disease is necessary in both men and women.

### Clinical implications

Considering the proven association between CVD and periodontal disease, early examination and treatment for periodontal disease patients may play an important role in preventing the occurrence of CVD, which needs to be highly valued in daily clinical work. Additionally, treatment of periodontal disease may prevent the occurrence of CVD, and may also improve the status of CVD to some degree. As shown by Vidal et al. (57), periodontal therapy was able to attenuate arterial stiffness and reduce circulating inflammatory markers. Moreover, periodontal treatment with subunit-microbial doses of doxycycline could increase the level of serum apolipoprotein A and high-density lipoprotein, reduce total cholesterol levels, and further reduce the risk of cardiovascular events (58, 59). As a result, periodontal disease therapy may be a therapeutic target for reduce the risk of CVD, and further relevant research is needed. However, it is interesting to note that periodontal pathogens are found in the interdental spaces even in young people with healthy periodontitis. The interdental health is thought to be closely related to cardiovascular diseases (60–62), therefore, the oral health of the interdental space should be promoted during adolescence to prevent periodontal disease and thus cardiovascular disease. Traditional daily methods to maintain oral health between teeth by destroying biofilms are still not effective enough. Studies have shown that using calibrated interdental brushes to clean teeth every day can reduce interdental bleeding, inhibit periodontal pathogens, and re-establish symbiotic microflora. and reduce interdental inflammation (63, 64). These findings suggest that adherence to interdental cleanliness may be an effective way to help maintain optimal oral health, thereby preventing the emergence of periodontal disease and ultimately reducing the risk of cardiovascular disease. In addition, one study had shown that daily use of mouthwash can also help prevent periodontal disease (65).

### Study limitations

There were still some limitations in present study. First, periodontal examination may be a more objective reflection of periodontal health than a self-reported diagnosis of periodontitis (66), but some of our included studies identified periodontal diseases by self-report. Second, some of the included studies were retrospective, which may have introduced recall bias, thus, further prospective studies are needed to confirm our results. Third, a high degree of heterogeneity was observed in our results due to the variation in the characteristics of the study population and study design. Finally, studies included in this meta-analysis are observational studies, however, observational studies cannot completely avoid some potential confusions, and the quality of evidence is not high.

## Conclusion

The findings of this study suggest that CVD is common in patients with periodontal disease. Periodontal disease is associated with an increased risk of CVD independent of sex. Further trials are required to assess the effect of periodontal intervention on the CVD incidence.

## Data availability statement

The datasets presented in this study can be found in online repositories. The names of the repository/repositories and accession number(s) can be found in the article/Supplementary material.

## Author contributions

QD, YL, and QH contributed to the study concept and design and revised the draft. QL, XY, JC, ML, and ZY performed the search strategy and contributed to database research, acquisition of data, and statistical analyses. All authors participated in data analysis, reviewed, and approved the final manuscript.

## References

[1] Manolis AA Manolis TA Melita H Manolis AS. Psoriasis and cardiovascular disease: the elusive link. *Int Rev Immunol.* (2019) 38:33–54. 10.1080/08830185.2018.1539084 30457023

[2] Colonia-García A Gutiérrez-Vélez M Duque-Duque A de Andrade CR. Possible association of periodontal disease with oral cancer and oral potentially malignant disorders: a systematic review. *Acta Odontol Scand.* (2020) 78:553–9. 10.1080/00016357.2020.1774076 32552160

[3] Tezal M Grossi SG Genco RJ. Is periodontitis associated with oral neoplasms? *J Periodontol.* (2005) 76:406–10. 10.1902/jop.2005.76.3.406 15857075

[4] Roth GA Mensah GA Johnson CO Addolorato G Ammirati E Baddour LM Global burden of cardiovascular diseases and risk factors, 1990-2019: update from the GBD 2019 study. *J Am Coll Cardiol.* (2020) 76:2982–3021. 33309175 10.1016/j.jacc.2020.11.010 PMC7755038

[5] Carrizales-Sepúlveda EF Ordaz-Farías A Vera-Pineda R Flores-Ramírez R. Periodontal disease, systemic inflammation and the risk of cardiovascular disease. *Heart Lung Circ.* (2018) 27:1327–34. 10.1016/j.hlc.2018.05.102 29903685

[6] Touil D Oualha L Douki N. Oral cancer: a major and growing public health problem towards a national policy of prevention and early detection in Tunisia. *Pan Afr Med J.* (2020) 37:291. 10.11604/pamj.2020.37.291.25448 33623628 PMC7881927

[7] Tezal M Sullivan MA Reid ME Marshall JR Hyland A Loree T Chronic periodontitis and the risk of tongue cancer. *Arch Otolaryngol Head Neck Surg.* (2007) 133:450–4. 10.1001/archotol.133.5.450 17515503

[8] Javed F Warnakulasuriya S. Is there a relationship between periodontal disease and oral cancer? A systematic review of currently available evidence. *Cri Rev Oncol Hematol.* (2016) 97:197–205. 10.1016/j.critrevonc.2015.08.018 26343577

[9] Khan SS Beach LB Yancy CW. Sex-based differences in heart failure: JACC focus seminar 7/7. *J Am Coll Cardiol.* (2022) 79:1530–41. 10.1016/j.jacc.2022.02.013 35422249

[10] Beale AL Meyer P Marwick TH Lam CSP Kaye DM. Sex differences in cardiovascular pathophysiology: why women are overrepresented in heart failure with preserved ejection fraction. *Circulation.* (2018) 138:198–205. 10.1161/CIRCULATIONAHA.118.034271 29986961

[11] Page MJ McKenzie JE Bossuyt PM Boutron I Hoffmann TC Mulrow CD The PRISMA 2020 statement: an updated guideline for reporting systematic reviews. *BMJ.* (2021) 372:n71. 10.1136/bmj.n71 33782057 PMC8005924

[12] Yan Z Liu Y Li W Zhao X Lin W Zhang J Liver fibrosis scores and prognosis in patients with cardiovascular diseases: a systematic review and meta-analysis. *Eur J Clin Invest.* (2022) 52:e13855. 10.1111/eci.13855 36001034

[13] Liu X Long C Xiong Q Chen C Ma J Su Y Association of angiotensin converting enzyme inhibitors and angiotensin II receptor blockers with risk of COVID-19, inflammation level, severity, and death in patients with COVID-19: a rapid systematic review and meta-analysis. *Clin Cardiol.* (2020). 10.1002/clc.23421 [Epub ahead of print]. 32757246 PMC7436520

[14] Morrison HI Ellison LF Taylor GW. Periodontal disease and risk of fatal coronary heart and cerebrovascular diseases. *J Cardiovasc Risk.* (1999) 6:7–11. 10.1177/204748739900600102 10197286

[15] Beck JD Eke P Lin D Madianos P Couper D Moss K Associations between IgG antibody to oral organisms and carotid intima-medial thickness in community-dwelling adults. *Atherosclerosis.* (2005) 183:342–8. 10.1016/j.atherosclerosis.2005.03.017 15893320

[16] Lee YL Hu HY Huang N Hwang DK Chou P Chu D. Dental prophylaxis and periodontal treatment are protective factors to ischemic stroke. *Stroke.* (2013) 44:1026–30. 10.1161/STROKEAHA.111.000076 23422085

[17] LaMonte MJ Genco RJ Hovey KM Wallace RB Freudenheim JL Michaud DS History of periodontitis diagnosis and edentulism as predictors of cardiovascular disease, stroke, and mortality in postmenopausal women. *J Am Heart Assoc.* (2017) 6:e004518. 10.1161/JAHA.116.004518 28356279 PMC5532989

[18] Joshy G Arora M Korda RJ Chalmers J Banks E. Is poor oral health a risk marker for incident cardiovascular disease hospitalisation and all-cause mortality? Findings from 172 630 participants from the prospective 45 and up study. *BMJ Open.* (2016) 6:e012386. 10.1136/bmjopen-2016-012386 27577588 PMC5013478

[19] Hansen GM Egeberg A Holmstrup P Hansen PR. Relation of periodontitis to risk of cardiovascular and all-cause mortality (from a Danish nationwide cohort study). *Am J Cardiol.* (2016) 118:489–93. 10.1016/j.amjcard.2016.05.036 27372888

[20] Chen DY Lin CH Chen YM Chen HH. Risk of atrial fibrillation or flutter associated with periodontitis: a nationwide, population-based, cohort study. *PLoS One.* (2016) 11:e0165601. 10.1371/journal.pone.0165601 27798703 PMC5087888

[21] Ahn YB Shin MS Byun JS Kim HD. The association of hypertension with periodontitis is highlighted in female adults: results from the Fourth Korea national health and nutrition examination survey. *J Clin Periodontol.* (2015) 42:998–1005. 10.1111/jcpe.12471 26461204

[22] Yu YH Chasman DI Buring JE Rose L Ridker PM. Cardiovascular risks associated with incident and prevalent periodontal disease. *J Clinical Periodontol.* (2015) 42:21–8. 10.1111/jcpe.12335 25385537 PMC4300240

[23] Andriankaja OM Genco RJ Dorn J Dmochowski J Hovey K Falkner KL Periodontal disease and risk of myocardial infarction: the role of gender and smoking. *Eur J Epidemiol.* (2007) 22:699–705. 10.1007/s10654-007-9166-6 17828467

[24] Batty GD Jung KJ Mok Y Lee SJ Back JH Lee S Oral health and later coronary heart disease: cohort study of one million people. *Eur J Prev Cardiol.* (2018) 25:598–605. 10.1177/2047487318759112 29461088 PMC5946673

[25] Beck J Garcia R Heiss G Vokonas PS Offenbacher S. Periodontal disease and cardiovascular disease. *J Periodontol.* (1996) 67:1123–37. 10.1902/jop.1996.67.10s.1123 29539797

[26] Choe H Kim YH Park JW Kim SY Lee SY Jee SH. Tooth loss, hypertension and risk for stroke in a Korean population. *Atherosclerosis.* (2009) 203:550–6. 10.1016/j.atherosclerosis.2008.07.017 19013571

[27] DeStefano F Anda RF Kahn HS Williamson DF Russell CM. Dental disease and risk of coronary heart disease and mortality. *Br Med J.* (1993) 306:688–91. 10.1136/bmj.306.6879.688 8471920 PMC1677081

[28] Dietrich T Jimenez M Kaye EAK Vokonas PS Garcia RI. Age-dependent associations between chronic periodontitis/edentulism and risk of coronary heart disease. *Circulation.* (2008) 117:1668–74. 18362228 10.1161/CIRCULATIONAHA.107.711507 PMC2582144

[29] Heitmann BL Gamborg M. Remaining teeth, cardiovascular morbidity and death among adult Danes. *Prev Med.* (2008) 47:156–60. 10.1016/j.ypmed.2008.04.007 18534671

[30] Howell TH Ridker PM Ajani UA Hennekens CH Christen WG. Periodontal disease and risk of subsequent cardiovascular disease in U.S. male physicians. *J Am Coll Cardiol.* (2001) 37:445–50. 10.1016/S0735-1097(00)01130-X 11216961

[31] Hung HC Joshipura KJ Colditz G Manson JE Rimm EB Speizer FE The association between tooth loss and coronary heart disease in men and women. *J Public Health Dent.* (2004) 64:209–15. 10.1111/j.1752-7325.2004.tb02755.x 15562943

[32] Hung HC Willett W Merchant A Rosner BA Ascherio A Joshipura KJ. Oral health and peripheral arterial disease. *Circulation.* (2003) 107:1152–7. 10.1161/01.CIR.0000051456.68470.C8 12615794

[33] Jimenez M Krall EA Garcia RI Vokonas PS Dietrich T. Periodontitis and incidence of cerebrovascular disease in men. *Ann Neurol.* (2009) 66:505–12. 10.1002/ana.21742 19847898 PMC2783821

[34] Joshipura KJ Hung HC Rimm EB Willett WC Ascherio A. Periodontal disease, tooth loss, and incidence of ischemic stroke. *Stroke.* (2003) 34:47–52. 10.1161/01.STR.0000052974.79428.0C 12511749

[35] Joshipura KJ Rimm EB Douglass CW Trichopoulos D Ascherio A Willett WC. Poor oral health and coronary heart disease. *J Dent Res.* (1996) 75:1631–6. 10.1177/00220345960750090301 8952614

[36] Noguchi S Toyokawa S Miyoshi Y Suyama Y Inoue K Kobayashi Y. Five-year follow-up study of the association between periodontal disease and myocardial infarction among Japanese male workers: my health up study. *J Public Health.* (2015) 37:605–11. 10.1093/pubmed/fdu076 25293424

[37] Rivas-Tumanyan S Spiegelman D Curhan GC Forman JP Joshipura KJ. Periodontal disease and incidence of hypertension in the health professionals follow-up study. *Am J Hypertens.* (2012) 25:770–6. 10.1038/ajh.2012.32 22476024 PMC3508690

[38] Senba T Kobayashi Y Inoue K Kaneto C Inoue M Toyokawa S The association between self-reported periodontitis and coronary heart disease–from my health up study. *J Occup Health.* (2008) 50:283–7. 10.1539/joh.L7066 18413975

[39] Tuominen R Reunanen A Paunio M Paunio I Aromaa A. Oral health indicators poorly predict coronary heart disease deaths. *J Dent Res.* (2003) 82:713–8. 10.1177/154405910308200911 12939356

[40] Dietrich T Jimenez M Krall Kaye EA Vokonas PS Garcia RI. Age-dependent associations between chronic periodontitis/edentulism and risk of coronary heart disease. *Circulation.* (2008) 117:1668–74. 10.1161/CIRCULATIONAHA.107.711507 18362228 PMC2582144

[41] Beck JD Offenbacher S. Systemic effects of periodontitis: epidemiology of periodontal disease and cardiovascular disease. *J Periodontol.* (2005) 76:2089–100. 10.1902/jop.2005.76.11-S.2089 16277581

[42] Atkins D Best D Briss PA Eccles M Falck-Ytter Y Flottorp S Grading quality of evidence and strength of recommendations. *BMJ.* (2004) 328:1490. 10.1136/bmj.328.7454.1490 15205295 PMC428525

[43] Larvin H Kang J Aggarwal VR Pavitt S Wu J. Risk of incident cardiovascular disease in people with periodontal disease: a systematic review and meta-analysis. *Clin Exp Dent Res.* (2021) 7:109–22. 10.1002/cre2.336 33124761 PMC7853902

[44] Lazureanu PC Popescu FG Stef L Focsa M Vaida MA Mihaila R. The Influence of periodontal disease on oral health quality of life in patients with cardiovascular disease: a cross-sectional observational single-center study. *Medicina.* (2022) 58:584. 10.3390/medicina58050584 35630001 PMC9144554

[45] Steven S Frenis K Oelze M Kalinovic S Kuntic M Bayo Jimenez MT Vascular inflammation and oxidative stress: major triggers for cardiovascular disease. *Oxid Med Cell Longev.* (2019) 2019:7092151. 10.1155/2019/7092151 31341533 PMC6612399

[46] Ndrepepa G. Myeloperoxidase - A bridge linking inflammation and oxidative stress with cardiovascular disease. *Clin Chim Acta.* (2019) 493:36–51. 10.1016/j.cca.2019.02.022 30797769

[47] Gu Y Lee H-M Sorsa T Salminen A Ryan ME Slepian MJ Non-antibacterial tetracyclines modulate mediators of periodontitis and atherosclerotic cardiovascular disease: a mechanistic link between local and systemic inflammation. *Pharmacol Res.* (2011) 64:573–9. 10.1016/j.phrs.2011.06.023 21771657

[48] Loos BG. Systemic markers of inflammation in periodontitis. *J Periodontol.* (2005) 76:2106–15. 10.1902/jop.2005.76.11-S.2106 16277583

[49] Stewart R West M. Increasing evidence for an association between periodontitis and cardiovascular disease. *Circulation.* (2016) 133:549–51. 10.1161/CIRCULATIONAHA.115.020869 26762522

[50] Aarabi G Heydecke G Seedorf U. Roles of oral infections in the pathomechanism of atherosclerosis. *Int J Mol Sci.* (2018) 19:1978. 10.3390/ijms19071978 29986441 PMC6073301

[51] Chun YH Chun KR Olguin D Wang HL. Biological foundation for periodontitis as a potential risk factor for atherosclerosis. *J Periodontal Res.* (2005) 40:87–95. 10.1111/j.1600-0765.2004.00771.x 15613084

[52] Khlgatian M Nassar H Chou HH Gibson FC III Genco CA. Fimbria-dependent activation of cell adhesion molecule expression in Porphyromonas gingivalis-infected endothelial cells. *Infect Immun.* (2002) 70:257–67. 10.1128/IAI.70.1.257-267.2002 11748191 PMC127610

[53] Srisuwantha R Shiheido Y Aoyama N Sato H Kure K Laosrisin N Porphyromonas gingivalis elevated high-mobility group box 1 levels after myocardial infarction in mice. *Int Heart J.* (2017) 58:762–8. 10.1536/ihj.16-500 28966323

[54] Shiheido Y Maejima Y Suzuki JI Aoyama N Kaneko M Watanabe R Porphyromonas gingivalis, a periodontal pathogen, enhances myocardial vulnerability, thereby promoting post-infarct cardiac rupture. *J Mol Cell Cardiol.* (2016) 99:123–37. 10.1016/j.yjmcc.2016.03.017 27079251

[55] Funaki S Tokutomi F Wada-Takahashi S Yoshino F Yoshida A Maehata Y Porphyromonas gingivalis infection modifies oral microcirculation and aortic vascular function in the stroke-prone spontaneously hypertensive rat (SHRSP). *Microb Pathog.* (2016) 92:36–42. 10.1016/j.micpath.2015.12.009 26724741

[56] Weissman S Sinh P Mehta TI Thaker RK Derman A Heiberger C Atherosclerotic cardiovascular disease in inflammatory bowel disease: the role of chronic inflammation. *World J Gastrointest Pathophysiol.* (2020) 11:104–13. 10.4291/wjgp.v11.i5.104 32832194 PMC7403753

[57] Vidal F Cordovil I Figueredo CM Fischer RG. Non-surgical periodontal treatment reduces cardiovascular risk in refractory hypertensive patients: a pilot study. *J Clin Periodontol.* (2013) 40:681–7. 10.1111/jcpe.12110 23639076

[58] Tüter G Serdar M Kurtiş B Walker SG Atak A Toyman U Effects of scaling and root planning and subantimicrobial dose doxycycline on gingival crevicular fluid levels of matrix metalloproteinase-8, -13 and serum levels of HsCRP in patients with chronic periodontitis. *J Periodontol.* (2010) 81:1132–9. 10.1902/jop.2010.090694 20370419

[59] Tüter G Kurtiş B Serdar M Aykan T Okyay K Yücel A Effects of scaling and root planning and sub-antimicrobial dose doxycycline on oral and systemic biomarkers of disease in patients with both chronic periodontitis and coronary artery disease. *J Clin Periodontol.* (2007) 34:673–81. 10.1111/j.1600-051X.2007.01104.x 17590156

[60] Teles R Wang CY. Mechanisms involved in the association between periodontal diseases and cardiovascular disease. *Oral Dis.* (2011) 17:450–61. 10.1111/j.1601-0825.2010.01784.x 21223455 PMC3373016

[61] Desvarieux M Demmer RT Jacobs DR Papapanou PN Sacco RL Rundek T. Changes in clinical and microbiological periodontal profiles relate to progression of carotid intima-media thickness: the oral infections and vascular disease epidemiology study. *J Am Heart Assoc.* (2013) 2:e000254. 10.1161/JAHA.113.000254 24166489 PMC3886779

[62] Li C Lv Z Shi Z Zhu Y Wu Y Li L Periodontal therapy for the management of cardiovascular disease in patients with chronic periodontitis. *Cochrane Database Syst Rev.* (2014) 8:CD009197. 10.1002/14651858.CD009197.pub2 25123257

[63] Bourgeois D Saliasi I Llodra JC Bravo M Viennot S Carrouel F. Efficacy of interdental calibrated brushes on bleeding reduction in adults: a 3-month randomized controlled clinical trial. *Eur J Oral Sci.* (2016) 124:566–71. 10.1111/eos.12302 27681016

[64] Bourgeois D Bravo M Llodra JC Inquimbert C Viennot S Dussart C Calibrated interdental brushing for the prevention of periodontal pathogens infection in young adults - a randomized controlled clinical trial. *Sci Rep.* (2019) 9:15127. 10.1038/s41598-019-51938-8 31641199 PMC6805917

[65] Saliasi I Llodra JC Bravo M Tramini P Dussart C Viennot S Effect of a toothpaste/mouthwash containing carica papaya leaf extract on interdental gingival bleeding: a randomized controlled trial. *Int J Environ Res Public Health.* (2018) 15:2660. 10.3390/ijerph15122660 30486374 PMC6313435

[66] LaMonte MJ Hovey KM Millen AE Genco RJ Wactawski-Wende J. Accuracy of self-reported periodontal disease in the women’s health initiative observational study. *J Periodontol.* (2014) 85:1006–18. 10.1902/jop.2013.130488 24354649 PMC6004791

[67] Moher D Liberati A Tetzlaff J Altman DG The PRISMA Group. Preferred reporting items for systematic reviews and meta-analyses: The PRISMA statement. *PLoS Med.* (2009) 6:e1000097. 10.1371/journal.pmed.1000097 19621072 PMC2707599

