# Supplementary material for: Periodontal disease is associated with the risk of cardiovascular disease independent of sex: A meta-analysis
Source: Front Cardiovasc Med. 2023 Feb 27;10:1114927. doi: 10.3389/fcvm.2023.1114927 (PMC10010192; doi:10.3389/fcvm.2023.1114927)
Supplement: Supplementary file 1 [file Data_Sheet_1.docx]

**Periodontal disease is associated with cardiovascular disease independent of sex: a meta-analysis**

**Supplementary Table S1: Article content checklist**

| **Section/topic** | | **#** | **Checklist item** | **Reported on page #** |
| --- | --- | --- | --- | --- |
| **TITLE** | | | |  |
| Title | | 1 | Identify the report as a systematic review, meta-analysis, or both. | 1 |
| **ABSTRACT** | | | |  |
| Structured summary | | 2 | Provide a structured summary including, as applicable: background; objectives; data sources; study eligibility criteria, participants, and interventions; study appraisal and synthesis methods; results; limitations; conclusions and implications of key findings; systematic review registration number. | 1 |
| **INTRODUCTION** | | | |  |
| Rationale | | 3 | Describe the rationale for the review in the context of what is already known. | 2 |
| Objectives | | 4 | Provide an explicit statement of questions being addressed with reference to participants, interventions, comparisons, outcomes, and study design (PICOS). | 2 |
| **METHODS** | | | |  |
| Protocol and registration | | 5 | Indicate if a review protocol exists, if and where it can be accessed (e.g., Web address), and, if available, provide registration information including registration number. | 3 |
| Eligibility criteria | | 6 | Specify study characteristics (e.g., PICOS, length of follow-up) and report characteristics (e.g., years considered, language, publication status) used as criteria for eligibility, giving rationale. | 4 |
| Information sources | | 7 | Describe all information sources (e.g., databases with dates of coverage, contact with study authors to identify additional studies) in the search and date last searched. | 4 |
| Search | | 8 | Present full electronic search strategy for at least one database, including any limits used, such that it could be repeated. | 4 |
| Study selection | | 9 | State the process for selecting studies (i.e., screening, eligibility, included in systematic review, and, if applicable, included in the meta-analysis). | 4 |
| Data collection process | | 10 | Describe method of data extraction from reports (e.g., piloted forms, independently, in duplicate) and any processes for obtaining and confirming data from investigators. | 5 |
| Data items | | 11 | List and define all variables for which data were sought (e.g., PICOS, funding sources) and any assumptions and simplifications made. | 5 |
| Risk of bias in individual studies | | 12 | Describe methods used for assessing risk of bias of individual studies (including specification of whether this was done at the study or outcome level), and how this information is to be used in any data synthesis. | 6 |
| Summary measures | | 13 | State the principal summary measures (e.g., risk ratio, difference in means). | 6 |
| Synthesis of results | | 14 | Describe the methods of handling data and combining results of studies, if done, including measures of consistency (e.g., I^2^) for each meta-analysis. | 7 |
| Risk of bias across studies | | 15 | Specify any assessment of risk of bias that may affect the cumulative evidence (e.g., publication bias, selective reporting within studies). | 7 |
| Additional analyses | | 16 | Describe methods of additional analyses (e.g., sensitivity or subgroup analyses, meta-regression), if done, indicating which were pre-specified. | 7 |
| **RESULTS** | | | |  |
| Study selection | 17 | | Give numbers of studies screened, assessed for eligibility, and included in the review, with reasons for exclusions at each stage, ideally with a flow diagram. | 7 |
| Study characteristics | 18 | | For each study, present characteristics for which data were extracted (e.g., study size, PICOS, follow-up period) and provide the citations. | 8 |
| Risk of bias within studies | 19 | | Present data on risk of bias of each study and, if available, any outcome level assessment (see item 12). | 9 |
| Results of individual studies | 20 | | For all outcomes considered (benefits or harms), present, for each study: (a) simple summary data for each intervention group (b) effect estimates and confidence intervals, ideally with a forest plot. | 9 |
| Synthesis of results | 21 | | Present results of each meta-analysis done, including confidence intervals and measures of consistency. | 10 |
| Risk of bias across studies | 22 | | Present results of any assessment of risk of bias across studies (see Item 15). | 10 |

**Supplementary Table S2**: **Detailed description of the search strategy**

| Datebase | Search | Query |
| --- | --- | --- |
| PubMed database | #1 | (((((((((((("Periodontal Disease"[Mesh]) OR (furcation defects)) OR (gingival diseases)) OR (peri-implantitis)) OR (periapical diseases)) OR (periodontal atrophy)) OR (periodontal cyst)) OR (periodontitis)) OR (tooth migration)) OR (periodontitis)) OR (tooth mobility)) OR (tooth loss)) |
|  | #2 | (((((((((((((((((((((((("Cardiovascular Diseases"[Mesh]) OR (cardiovascular disease)) OR (cardiovascular diseases)) OR (CVD)) OR (coronary heart disease)) OR (CHD)) OR (coronary artery disease)) OR (CAD)) OR (myocardial infarction)) OR (MI)) OR (coronary arteriosclerosis)) OR (heart attack)) OR (heart failure)) OR (HF)) OR (heart decompensation)) OR (atrial fibrillation)) OR (AF)) OR (sudden cardiac death)) OR (SCD)) OR (arrhythmia)) OR (cardiomyopathy)) OR (hypertrophic cardiomyopathy)) OR (HCM)) OR (dilated cardiomyopathy)) OR (DCM)) |
|  | #3 | #1 AND #2 |
| Embase database | #1 | ‘Periodontal Disease’/exp |
|  | #2 | ‘furcation defects’ OR ‘gingival diseases’ OR ‘peri-implantitis’ OR ‘periapical diseases’ OR ‘periodontal atrophy’ OR ‘periodontal cyst’ OR ‘periodontitis’ OR ‘tooth migration’ OR ‘periodontitis’ OR ‘tooth mobility’ OR ‘tooth loss’ |
|  | #3 | 'Cardiovascular Diseases'/exp |
|  | #4 | ‘cardiovascular disease’ OR ‘cardiovascular diseases’ OR ‘CVD’ OR ‘coronary heart disease’ OR ‘CHD’ OR ‘coronary artery disease’ OR ‘CAD’ OR ‘myocardial infarction’ OR ‘MI’ OR ‘coronary arteriosclerosis’ OR ‘heart attack’ OR ‘heart failure’ OR ‘HF’ OR ‘heart decompensation’ OR ‘atrial fibrillation’ OR ‘AF’ OR ‘sudden cardiac death’ OR ‘SCD’ OR ‘arrhythmia’ OR ‘cardiomyopathy’ OR ‘hypertrophic cardiomyopathy’ OR ‘HCM’ OR ‘dilated cardiomyopathy’ OR ‘DCM’ |
|  | #5 | #1 OR #2 |
|  | #6 | #3 OR #4 |
|  | #7 | #5 AND #6 |
| Cochrane library | #1 | MeSH descriptor: [Periodontal Disease] explode all trees |
|  | #2 | (Periodontal Disease):ti,ab,kw |
|  | #3 | MeSH descriptor: [Cardiovascular Diseases] explode all trees |
|  | #4 | (Cardiovascular Diseases):ti,ab,kw |
|  | #5 | #1 OR #2 |
|  | #6 | #3 OR #4 |
|  | #7 | #5 AND #6 |

**Embase**

| **Search** | **Query** |
| --- | --- |
| #1 | 'cardiovascular disease':ab,ti OR 'cardiovascular abnormalities':ab,ti OR 'congenital blood vessel malformation':ab,ti OR 'cardiovascular infection':ab,ti OR 'bacterial endocarditis':ab,ti OR 'cardiovascular syphilis':ab,ti OR tuberculosis:ab,ti OR 'heart disease':ab,ti OR 'heart arrhythmia':ab,ti OR 'carcinoid syndrome':ab,ti OR 'heart muscle conduction disturbance':ab,ti OR 'high output heart failure':ab,ti OR 'forward heart failure':ab,ti OR 'heart tamponade':ab,ti OR 'cardiomegaly':ab,ti OR cardiomyopathy:ab,ti OR cardiotoxicity:ab,ti OR endocarditis:ab,ti OR 'heart aneurysm':ab,ti OR 'heart arrest':ab,ti OR 'congenital heart malformation':ab,ti OR 'heart failure':ab,ti OR 'heart tumor':ab,ti OR 'heart rupture':ab,ti OR 'valvular heart disease':ab,ti OR 'heart muscle ischemia':ab,ti OR 'stunned heart muscle':ab,ti OR 'pericardial effusion':ab,ti OR pericarditis:ab,ti OR pneumopericardium:ab,ti OR 'post-cardiac arrest syndrome':ab,ti OR 'postpericardiotomy syndrome':ab,ti OR 'pulmonary heart disease':ab,ti OR 'rheumatic heart disease':ab,ti OR 'ventricular dysfunction':ab,ti OR 'ventricular outflow obstruction':ab,ti OR 'pregnancy complications, cardiovascular':ab,ti OR 'embolism, amniotic fluid':ab,ti OR 'vascular diseases':ab,ti OR aneurysm:ab,ti OR angiodysplasia:ab,ti OR 'angioneurotic edema':ab,ti OR 'angiomatosis +':ab,ti OR 'aortic diseases +':ab,ti OR 'arterial occlusive diseases +':ab,ti OR 'arteriovenous malformations +':ab,ti OR 'capillary leak syndrome':ab,ti OR 'cerebrovascular disease':ab,ti OR 'colitis, ischemic':ab,ti OR 'compartment syndromes':ab,ti OR 'diabetic angiopathy':ab,ti OR (embolism:ab,ti AND thrombosis:ab,ti) OR 'hand-arm vibration syndrome':ab,ti OR hemorrhoid:ab,ti OR 'hemostatic disorders':ab,ti OR 'hepatic veno-occlusive disease':ab,ti OR hyperemia:ab,ti OR hypertension:ab,ti OR hypotension:ab,ti OR 'mesenteric ischemia':ab,ti OR 'myocardial ischemia':ab,ti OR 'optic neuropathy, ischemic':ab,ti OR 'peliosis hepatis':ab,ti OR 'peripheral vascular diseases':ab,ti OR prehypertension:ab,ti OR 'pulmonary veno-occlusive disease':ab,ti OR 'reperfusion injury':ab,ti OR 'retinal vein occlusion':ab,ti OR 'scimitar syndrome':ab,ti OR 'spinal cord vascular diseases':ab,ti OR 'spleen infarction':ab,ti OR 'stenosis, pulmonary vein':ab,ti OR 'superior vena cava syndrome':ab,ti OR telangiectasis:ab,ti OR 'thoracic outlet syndrome':ab,ti OR varicocele:ab,ti OR 'varicose veins':ab,ti OR 'vascular fistula':ab,ti OR 'vascular neoplasms':ab,ti OR 'vascular system injuries':ab,ti OR vasculitis:ab,ti OR vasoplegia:ab,ti OR 'venous insufficiency':ab,ti |
| #2 | 'periodontal disease':ab,ti OR 'furcation defects':ab,ti OR 'gingival diseases':ab,ti OR 'gingival hemorrhage':ab,ti OR 'gingival neoplasms':ab,ti OR 'gingival overgrowth':ab,ti OR gingivitis:ab,ti OR 'granuloma, giant cell':ab,ti OR pericoronitis:ab,ti OR 'peri-implantitis':ab,ti OR 'periapical diseases':ab,ti OR 'radicular cyst':ab,ti OR 'periodontal atrophy':ab,ti OR 'alveolar bone loss':ab,ti OR 'gingival recession':ab,ti OR 'periodontal attachment loss':ab,ti OR 'periodontal cyst':ab,ti OR 'periodontitis':ab,ti OR 'aggressive periodontitis':ab,ti OR 'chronic periodontitis':ab,ti OR 'periapical periodontitis':ab,ti OR 'periodontal abscess':ab,ti OR 'periodontal pocket':ab,ti OR 'tooth loss':ab,ti OR 'tooth migration':ab,ti OR 'mesial movement of teeth':ab,ti OR 'tooth mobility':ab,ti |
| #3 | #1 AND #2 |

**Cochrane**

| **Search** | **Query** |
| --- | --- |
| #1 | (Periodontal Diseases):ti,ab,kw OR (Furcation Defects):ti,ab,kw OR (Gingival Diseases):ti,ab,kw OR (Gingival Hemorrhage):ti,ab,kw OR (Gingival Neoplasms):ti,ab,kw (Word variations have been searched) |
| #2 | (Gingival Overgrowth):ti,ab,kw OR (Gingival Recession):ti,ab,kw OR (Gingivitis):ti,ab,kw OR (Granuloma, Giant Cell):ti,ab,kw OR (Pericoronitis):ti,ab,kw (Word variations have been searched) |
| #3 | (Peri-Implantitis):ti,ab,kw OR (Periapical Diseases):ti,ab,kw OR (Periapical Periodontitis):ti,ab,kw OR ((Radicular Cyst)):ti,ab,kw OR (Periodontal Atrophy):ti,ab,kw (Word variations have been searched) |
| #4 | (Alveolar Bone Loss):ti,ab,kw OR (Gingival Recession):ti,ab,kw OR (Periodontal Attachment Loss):ti,ab,kw OR (Periodontal Cyst):ti,ab,kw OR (Periodontitis):ti,ab,kw (Word variations have been searched) |
| #5 | (Aggressive Periodontitis):ti,ab,kw OR (Chronic Periodontitis):ti,ab,kw OR (Periapical Periodontitis):ti,ab,kw OR (Periodontal Abscess):ti,ab,kw OR (Periodontal Pocket):ti,ab,kw (Word variations have been searched) |
| #6 | (Tooth Loss):ti,ab,kw OR (Tooth Migration):ti,ab,kw OR (Mesial Movement of Teeth):ti,ab,kw OR (Tooth Mobility):ti,ab,kw (Word variations have been searched) |
| #7 | #1 OR #2 OR #3 OR #4 OR #5 OR #6 |
| #8 | (Cardiovascular Diseases):ti,ab,kw OR (Cardiovascular Abnormalities):ti,ab,kw OR (Heart Defects, Congenital):ti,ab,kw OR ((Vascular Malformations):ti,ab,kw OR (Cardiovascular Infections):ti,ab,kw (Word variations have been searched) |
| #9 | (Endocarditis, Bacterial ):ti,ab,kw OR (Syphilis, Cardiovascular):ti,ab,kw OR (Tuberculosis, Cardiovascular ):ti,ab,kw OR (Heart Diseases):ti,ab,kw OR (Arrhythmias, Cardiac):ti,ab,kw (Word variations have been searched) |
| #10 | (Carcinoid Heart Disease ):ti,ab,kw OR (Cardiac Conduction System Disease ):ti,ab,kw OR (Cardiac Output, High ):ti,ab,kw OR (Cardiac Output, Low):ti,ab,kw OR (Cardiac Tamponade):ti,ab,kw (Word variations have been searched) |
| #11 | (Cardiomegaly ):ti,ab,kw OR (Cardiomyopathies ):ti,ab,kw OR (Cardiotoxicity ):ti,ab,kw OR (Endocarditis):ti,ab,kw OR (Heart Aneurysm):ti,ab,kw (Word variations have been searched) |
| #12 | (Heart Arrest ):ti,ab,kw OR (Heart Defects, Congenital ):ti,ab,kw OR (Heart Failure):ti,ab,kw OR (Heart Neoplasms ):ti,ab,kw OR (Heart Rupture):ti,ab,kw (Word variations have been searched) |
| #13 | (Heart Valve Diseases ):ti,ab,kw OR (Myocardial Ischemia ):ti,ab,kw OR (Myocardial Stunning):ti,ab,kw OR (Pericardial Effusion ):ti,ab,kw OR (Pericarditis):ti,ab,kw (Word variations have been searched) |
| #14 | (Pneumopericardium ):ti,ab,kw OR (Post-Cardiac Arrest Syndrome ):ti,ab,kw OR (Postpericardiotomy Syndrome):ti,ab,kw OR (Pulmonary Heart Disease ):ti,ab,kw OR (Rheumatic Heart Disease):ti,ab,kw (Word variations have been searched) |
| #15 | (Ventricular Dysfunction ):ti,ab,kw OR (Ventricular Outflow Obstruction ):ti,ab,kw OR((Pregnancy Complications, Cardiovascular):ti,ab,kw OR (Embolism, Amniotic Fluid ):ti,ab,kw OR (Vascular Diseases):ti,ab,kw (Word variations have been searched) |
| #16 | (Aneurysm):ti,ab,kw OR ((Angiodysplasia ):ti,ab,kw OR ("angioedema-urticaria"):ti,ab,kw OR (Angiomatosis ):ti,ab,kw OR (Aortic Diseases)):ti,ab,kw (Word variations have been searched) |
| #17 | (Arterial Occlusive Diseases):ti,ab,kw OR (Arteriovenous Malformations ):ti,ab,kw OR (Capillary Leak Syndrome):ti,ab,kw OR (Cerebrovascular Disorders):ti,ab,kw OR (Colitis, Ischemic):ti,ab,kw (Word variations have been searched) |
| #18 | (Compartment Syndromes):ti,ab,kw OR (Diabetic Angiopathies):ti,ab,kw OR (Embolism and Thrombosis):ti,ab,kw OR (Hand-Arm Vibration Syndrome):ti,ab,kw OR (Hemorrhoids):ti,ab,kw (Word variations have been searched) |
| #19 | (Hemostatic Disorders):ti,ab,kw OR (Hepatic Veno-Occlusive Disease):ti,ab,kw OR (Hyperemia):ti,ab,kw OR (Hypertension):ti,ab,kw OR (Hypotension):ti,ab,kw (Word variations have been searched) |
| #20 | (Mesenteric Ischemia):ti,ab,kw OR (Myocardial Ischemia):ti,ab,kw OR (Optic Neuropathy, Ischemic):ti,ab,kw OR (Peliosis Hepatis):ti,ab,kw OR (Peripheral Vascular Diseases):ti,ab,kw (Word variations have been searched) |
| #21 | (Prehypertension):ti,ab,kw OR (Pulmonary Veno-Occlusive Disease):ti,ab,kw OR (Reperfusion Injury):ti,ab,kw OR (Retinal Vein Occlusion):ti,ab,kw OR (Scimitar Syndrome):ti,ab,kw (Word variations have been searched) |
| #22 | (Spinal Cord Vascular Diseases):ti,ab,kw OR (Splenic Infarction):ti,ab,kw OR (Stenosis, Pulmonary Vein):ti,ab,kw OR (Superior Vena Cava Syndrome):ti,ab,kw OR (Telangiectasis):ti,ab,kw (Word variations have been searched) |
| #23 | (Thoracic Outlet Syndrome):ti,ab,kw OR (Varicocele):ti,ab,kw OR (Varicose Veins):ti,ab,kw OR (Vascular Fistula):ti,ab,kw OR (Vascular Neoplasms):ti,ab,kw (Word variations have been searched) |
| #24 | (Vascular System Injuries):ti,ab,kw OR (Vasculitis):ti,ab,kw OR (Vasoplegia):ti,ab,kw OR (Venous Insufficiency):ti,ab,kw (Word variations have been searched) |
| #25 | #8 OR #9 OR #10 OR #11 OR #12 OR #13 #14 OR #15 OR #16 OR #17 OR #18 OR #19 #20 OR #21 OR #22 OR #23 OR #24 |
| #26 | #7 AND #25 |

**Supplementary Table S3: Studies excluded (n=55) with reasons**

| **Studies excluded** | **Reasons** |
| --- | --- |
| Gong 2017^1^ | A meta-analysis |
| Grau 2004^2^ | The sample size is insufficient |
| Hada^3^ | The sample size is insufficient |
| Bal 2015^4^ | The sample size is insufficient |
| Arbes 1999^5^ | Can't extract accurate data |
| Buhlin 2002^6^ | Can't extract accurate data |
| Alade 2018^7^ | A meta-analysis |
| Alim 2020^7^ | The sample size is insufficient |
| Aarabi 2015^8^ | The sample size is insufficient |
| Abou-Raya 2008^9^ | Can't extract accurate data |
| Beck 2000^10^ | The sample size is insufficient |
| Cueto 2005^11^ | The sample size is insufficient |
| Darnaud2015 | Can't extract accurate data |
| Holmlund 2017^12^ | Can't extract accurate data |
| Hujoel 2001^13^ | Unable extract accurate data |
| Hujoel 2002^14^ | Can't extract accurate data |
| Dorn 2010^15^ | Unable extract accurate data |
| Kim 2010^16^ | Can't extract accurate data |
| Kodovazenitis 2014^17^ | Unable extract accurate data |
| Koppolu 2013^18^ | Can't extract accurate data |
| Lee 2019^19^ | Unable extract accurate data |
| Lee 2006^20^ | Unable extract accurate data |
| Loesche 1998^21^ | Can't extract accurate data |
| Pradeep 2010^22^ | Unable extract accurate data |
| Pray 2014^23^ | Unable extract accurate data |
| Pussinen 2004^24^ | The sample size is insufficient |
| Pussinen 2003^25^ | The sample size is insufficient |
| Rastogi 2012^26^ | Unable extract accurate data |
| Renvert 2010^27^ | The sample size is insufficient |
| Ryden 2016^28^ | The sample size is insufficient |
| Sen 2018^29^ | Unable extract accurate data |
| Tu 2017^30^ | The sample size is insufficient |
| Zagaria 2016^31^ | Unable extract accurate data |
| Joshy 2016^32^ | Unable extract accurate data |
| Elter 2004^33^ | The sample size is insufficient |
| Higashi 2009^34^ | Can't extract accurate data |
| Jacobs 2007^35^ | Can't extract accurate data |
| Liljestrand 2015^36^ | Unable extract accurate data |
| Loesche 1996^37^ | Can't extract accurate data |
| Montebugnoli 2005^38^ | Can't extract accurate data |
| Barilli 2006^39^ | Unable extract accurate data |
| Barnett 2009^40^ | Can't extract accurate data |
| Bawankar 2021^41^ | Can't extract accurate data |
| Behle 2006^42^ | The sample size is insufficient |
| Belinga 2018^43^ | The sample size is insufficient |
| Chen 2009^44^ | Unable extract accurate data |
| Choi 2015^45^ | The sample size is insufficient |
| Chou 2015^46^ | Can't extract accurate data |
| Da Venezia 2021^47^ | The sample size is insufficient |
| Dorfer 2004^48^ | The sample size is insufficient |
| Homlund 2011^49^ | Can't extract accurate data |
| Joshipura 2005^50^ | The sample size is insufficient |
| Soory 2010^51^ | Can't extract accurate data |
| Aminzadeh 2013^52^ | The sample size is insufficient |
| Lee 2015^53^ | The type of research is incorrect |
| Lee 2017^54^ | The type of research is incorrect |
| Alhadainy 2021^55^ | Can't extract accurate data |

1. Gong, Q.; Janowski, M.; Luo, M.; Wei, H.; Chen, B.; Yang, G.; Liu, L., Efficacy and Adverse Effects of Atropine in Childhood Myopia: A Meta-analysis. *JAMA Ophthalmol* **2017,** *135* (6), 624-630.

2. Grau, A. J.; Becher, H.; Ziegler, C. M.; Lichy, C.; Buggle, F.; Kaiser, C.; Lutz, R.; Bültmann, S.; Preusch, M.; Dörfer, C. E., Periodontal disease as a risk factor for ischemic stroke. *Stroke* **2004,** *35* (2), 496-501.

3. Hada, D. S.; Garg, S.; Ramteke, G. B.; Ratre, M. S., Effect of Non-Surgical Periodontal Treatment on Clinical and Biochemical Risk Markers of Cardiovascular Disease: a Randomized Trial. *Journal of periodontology* **2015,** *86* (11), 1201‐1211.

4. Bal, M. V.; Olgun, A.; Abaslı, D.; Özdemir, A.; Kürşaklıoğlu, H.; Göktepe, A. S.; Kurt, Í., The effect of nonsurgical periodontal treatment on serum and saliva chitotriosidase activities in patients with periodontitis and coronary artery disease. *Therapeutics and clinical risk management* **2015,** *11*, 53-8.

5. Arbes S.J, Jr.; Slade, G. D.; Beck, J. D., Association between extent of periodontal attachment loss and self-reported history of heart attack: An analysis of NHANES III data. *Journal of Dental Research* **1999,** *78* (12), 1777-1782.

6. Bokhari, S. A. H.; Khan, A. A.; Tatakis, D. N.; Azhar, M.; Hanif, M.; Izhar, M., Non-surgical periodontal therapy lowers serum inflammatory markers: A pilot study. *Journal of Periodontology* **2009,** *80* (10), 1574-1580.

7. Alade, G. O.; Ayanbadejo, P. O.; Umeizudike, K. A.; Ajuluchukwu, J. N., Association of Elevated C-Reactive Protein with Severe Periodontitis in Hypertensive Patients in Lagos, Nigeria: A Pilot Study. *Contemporary clinical dentistry* **2018,** *9* (Suppl 1), S95-s99.

8. Aarabi, G.; Eberhard, J.; Reissmann, D. R.; Heydecke, G.; Seedorf, U., Interaction between periodontal disease and atherosclerotic vascular disease--Fact or fiction? *Atherosclerosis* **2015,** *241* (2), 555-60.

9. Abou-Raya, S.; Abou-Raya, A.; Naim, A.; Abuelkheir, H., Rheumatoid arthritis, periodontal disease and coronary artery disease. *Clin Rheumatol* **2008,** *27* (4), 421-7.

10. Beck, J. D.; Slade, G.; Offenbacher, S., Oral disease, cardiovascular disease and systemic inflammation. *Periodontol 2000* **2000,** *23*, 110-20.

11. Cueto, A.; Mesa, F.; Bravo, M.; Ocaña-Riola, R., Periodontitis as risk factor for acute myocardial infarction. A case control study of Spanish adults. *Journal of Periodontal Research* **2005,** *40* (1), 36-42.

12. Holmlund, A.; Lampa, E.; Lind, L., Oral health and cardiovascular disease risk in a cohort of periodontitis patients. *Atherosclerosis* **2017,** *262*, 101-106.

13. Hujoel, P. P.; Drangsholt, M.; Spiekerman, C.; Derouen, T. A., Examining the link between coronary heart disease and the elimination of chronic dental infections. *Journal of the American Dental Association (1939)* **2001,** *132* (7), 883-889.

14. Hujoel, P. P.; Drangsholt, M.; Spiekerman, C.; DeRouen, T. A., Periodontitis-systemic disease associations in the presence of smoking--causal or coincidental? *Periodontol 2000* **2002,** *30*, 51-60.

15. Dorn, J. M.; Genco, R. J.; Grossi, S. G.; Falkner, K. L.; Hovey, K. M.; Iacoviello, L.; Trevisan, M., Periodontal disease and recurrent cardiovascular events in survivors of myocardial infarction (MI): The Western New York acute MI study. *Journal of Periodontology* **2010,** *81* (4), 502-511.

16. Kim, H. D.; Sim, S. J.; Moon, J. Y.; Hong, Y. C.; Han, D. H., Association between periodontitis and hemorrhagic stroke among Koreans: A case-control study. *Journal of Periodontology* **2010,** *81* (5), 658-665.

17. Kodovazenitis, G.; Pitsavos, C.; Papadimitriou, L.; Vrotsos, I. A.; Stefanadis, C.; Madianos, P. N., Association between periodontitis and acute myocardial infarction: a case-control study of a nondiabetic population. *Journal of periodontal research* **2014,** *49* (2), 246-252.

18. Koppolu, P.; Durvasula, S.; Palaparthy, R.; Rao, M.; Sagar, V.; Reddy, S. K.; Lingam, S., Estimate of CRP and TNF-alpha level before and after periodontal therapy in cardiovascular disease patients. *Pan African medical journal* **2013,** *15*, 92.

19. Lee, H. J.; Choi, E. K.; Park, J. B.; Han, K. D.; Oh, S., Tooth Loss Predicts Myocardial Infarction, Heart Failure, Stroke, and Death. *Journal of dental research* **2019,** *98* (2), 164-170.

20. Lee, H. J.; Garcia, R. I.; Janket, S. J.; Jones, J. A.; Mascarenhas, A. K.; Scott, T. E.; Nunn, M. E., The association between cumulative periodontal disease and stroke history in older adults. *The Journal of periodontology* **2006,** *77* (10), 1744-54.

21. Loesche, W. J.; Schork, A.; Terpenning, M. S.; Chen, Y. M.; Dominguez, B. L.; Grossman, N., Assessing the relationship between dental disease and coronary heart disease in elderly U.S. veterans. *J Am Dent Assoc* **1998,** *129* (3), 301-11.

22. Pradeep, A. R.; Hadge, P.; Arjun Raju, P.; Shetty, S. R.; Shareef, K.; Guruprasad, C. N., Periodontitis as a risk factor for cerebrovascular accident: A case-control study in the Indian population. *Journal of Periodontal Research* **2010,** *45* (2), 223-228.

23. Pray, W. S.; Pray, G. E., The link between Periodontitis and cardiovascular disease. 2014; Vol. 39, pp 15-17.

24. Pussinen, P. J.; Alfthan, G.; Rissanen, H.; Reunanen, A.; Asikainen, S.; Knekt, P., Antibodies to periodontal pathogens and stroke risk. *Stroke* **2004,** *35* (9), 2020-2023.

25. Pussinen, P. J.; Jousilahti, P.; Alfthan, G.; Palosuo, T.; Asikainen, S.; Salomaa, V., Antibodies to periodontal pathogens are associated with coronary heart disease. *Arteriosclerosis, thrombosis, and vascular biology* **2003,** *23* (7), 1250‐1254.

26. Rastogi, P.; Singhal, R.; Sethi, A.; Agarwal, A.; Singh, V. K.; Sethi, R., Assessment of the effect of periodontal treatment in patients with coronary artery disease : A pilot survey. *J Cardiovasc Dis Res* **2012,** *3* (2), 124-7.

27. Renvert, S.; Ohlsson, O.; Pettersson, T.; Persson, G. R., Periodontitis: A future risk of acute coronary syndrome? A follow-up study over 3 years. *Journal of Periodontology* **2010,** *81* (7), 992-1000.

28. Rydén, L.; Buhlin, K.; Ekstrand, E.; De Faire, U.; Gustafsson, A.; Holmer, J.; Kjellström, B.; Lindahl, B.; Norhammar, A.; Nygren, Å.; Näsman, P.; Rathnayake, N.; Svenungsson, E.; Klinge, B., Periodontitis Increases the Risk of a First Myocardial Infarction: A Report from the PAROKRANK Study. *Circulation* **2016,** *133* (6), 576-583.

29. Sen, S.; Giamberardino, L. D.; Moss, K.; Morelli, T.; Rosamond, W. D.; Gottesman, R. F.; Beck, J.; Offenbacher, S., Periodontal Disease, Regular Dental Care Use, and Incident Ischemic Stroke. *Stroke* **2018,** *49* (2), 355-362.

30. Tu, Y. K.; Galobardes, B.; Smith, G. D.; McCarron, P.; Jeffreys, M.; Gilthorpe, M. S., Associations between tooth loss and mortality patterns in the Glasgow Alumni Cohort. *Heart* **2007,** *93* (9), 1098-1103.

31. Zagaria, M. A. E., Periodontitis: Risk for atherosclerotic vascular disease? 2016; Vol. 41, pp 9-11.

32. Joshy, G.; Arora, M.; Korda, R. J.; Chalmers, J.; Banks, E., Is poor oral health a risk marker for incident cardiovascular disease hospitalisation and all-cause mortality? Findings from 172 630 participants from the prospective 45 and up study. *BMJ Open* **2016,** *6* (8).

33. Elter, J. R.; Champagne, C. M. E.; Offenbacher, S.; Beck, J. D., Relationship of periodontal disease and tooth loss to prevalence of coronary heart disease. *Journal of Periodontology* **2004,** *75* (6), 782-790.

34. Higashi, Y.; Goto, C.; Hidaka, T.; Soga, J.; Nakamura, S.; Fujii, Y.; Hata, T.; Idei, N.; Fujimura, N.; Chayama, K.; et al., Oral infection-inflammatory pathway, periodontitis, is a risk factor for endothelial dysfunction in patients with coronary artery disease. *Atherosclerosis* **2009,** *206* (2), 604‐610.

35. Jacobs Jr, D. R.; Crow, R. S., Subclinical cardiovascular disease markers applicable to studies of oral health: Multiethnic study of atherosclerosis. 2007; Vol. 1098, pp 269-287.

36. Liljestrand, J. M.; Havulinna, A. S.; Paju, S.; Männistö, S.; Salomaa, V.; Pussinen, P. J., Missing Teeth Predict Incident Cardiovascular Events, Diabetes, and Death. *Journal of dental research* **2015,** *94* (8), 1055-1062.

37. Loesche, W. J., Microbiology of Dental Decay and Periodontal Disease. In *Medical Microbiology*, Baron, S., Ed. University of Texas Medical Branch at Galveston

Copyright © 1996, The University of Texas Medical Branch at Galveston.: Galveston (TX), 1996.

38. Montebugnoli, L.; Servidio, D.; Miaton, R. A.; Prati, C.; Tricoci, P.; Melloni, C.; Melandri, G., Periodontal health improves systemic inflammatory and haemostatic status in subjects with coronary heart disease. *J Clin Periodontol* **2005,** *32* (2), 188-92.

39. Barilli, A. L. A.; Passos, A. D. C.; Marin-Neto, J. A.; Franco, L. J., Priodontal disease in patients with ischemic coronary atherosclerosis at a university hospital. *Arquivos Brasileiros de Cardiologia* **2006,** *87* (6), 635-640.

40. Barnett, A. H., Periodontal disease and general health: Implications for cardiovascular disease and diabetes. *Practical Diabetes International* **2009,** *26* (1), 31-33.

41. Bawankar, P. V.; Kolte, A. P.; Kolte, R. A., Assessment of knowledge, awareness, and attitude among patients with cardiovascular disease about its association with chronic periodontitis. *J Indian Soc Periodontol* **2021,** *25* (2), 156-161.

42. Behle, J. H.; Papapanou, P. N., Periodontal infections and atherosclerotic vascular disease: an update. *Int Dent J* **2006,** *56* (4 Suppl 1), 256-62.

43. Belinga, L. E. E.; Ngan, W. B.; Lemougoum, D.; Nlo'o, A.; Bongue, B.; Ngono, A.; Mandengue, S. H.; Sembene, M., Association between periodontal diseases and cardiovascular diseases in Cameroon. *J Public Health Afr* **2018,** *9* (1), 761.

44. Chen, Y. W.; Nagasawa, T.; Wara-Aswapati, N.; Ushida, Y.; Wang, D.; Takeuchi, Y.; Kobayashi, H.; Umeda, M.; Inoue, Y.; Iwai, T.; Ishikawa, I.; Izumi, Y., Association between periodontitis and anti-cardiolipin antibodies in Buerger disease. *J Clin Periodontol* **2009,** *36* (10), 830-5.

45. Choi, H. M.; Han, K.; Park, Y. G.; Park, J. B., Associations Among Oral Hygiene Behavior and Hypertension Prevalence and Control: The 2008 to 2010 Korea National Health and Nutrition Examination Survey. *Journal of periodontology* **2015,** *86* (7), 866-873.

46. Chou, S. H.; Tung, Y. C.; Lin, Y. S.; Wu, L. S.; Lin, C. P.; Liou, E. J.; Chang, C. J.; Kung, S.; Chu, P. H., Major Adverse Cardiovascular Events in Treated Periodontitis: A Population-Based Follow-Up Study from Taiwan. *PLoS One* **2015,** *10* (6), e0130807.

47. Da Venezia, C.; Hussein, N.; Hernández, M.; Contreras, J.; Morales, A.; Valdés, M.; Rojas, F.; Matamala, L.; Hernández-Ríos, P., Assessment of Cardiovascular Risk in Women with Periodontal Diseases According to C-reactive Protein Levels. *Biomolecules* **2021,** *11* (8).

48. Dörfer, C. E.; Becher, H.; Ziegler, C. M.; Kaiser, C.; Lutz, R.; Jörss, D.; Lichy, C.; Buggle, F.; Bültmann, S.; Preusch, M.; Grau, A. J., The association of gingivitis and periodontitis with ischemic stroke. *J Clin Periodontol* **2004,** *31* (5), 396-401.

49. Holmlund, A.; Lind, L., Markers of periodontal disease predict myocardial infarction, stroke and heart failure differently in a cohort of 7999 subjects. *Circulation* **2011,** *124* (21).

50. Joshipura, K. J.; Ritchie, C., Can the relation between tooth loss and chronic disease be explained by socio-economic status? *Eur J Epidemiol* **2005,** *20* (3), 203-4.

51. Soory, M., Association of periodontitis with rheumatoid arthritis and atherosclerosis: Novel paradigms in etiopathogeneses and management? 2010; Vol. 2, pp 1-16.

52. Aminzadeh, A.; Ahmadi, M.; Hosseini, S. M., Relation between Oral Health Status and Electrocardiogram ST Segment Changes in a Group of Patients with Myocardial Infarction. *Journal of dental research, dental clinics, dental prospects* **2013,** *7* (3), 169-73.

53. Lee, J. H.; Lee, J. S.; Park, J. Y.; Choi, J. K.; Kim, D. W.; Kim, Y. T.; Choi, S. H., Association of lifestyle-related comorbidities with periodontitis: A nationwide cohort study in Korea. *Medicine (United States)* **2015,** *94* (37).

54. Lee, J. H.; Oh, J. Y.; Youk, T. M.; Jeong, S. N.; Kim, Y. T.; Choi, S. H., Association between periodontal disease and non-communicable diseases: A 12-year longitudinal health-examinee cohort study in South Korea. *Medicine (United States)* **2017,** *96* (26).

55. Alhadainy, H. A.; Keefe, T.; Abdel-Karim, A. H.; Abdulrab, S.; Halboub, E., Association between dental diseases and history of stroke in the United States. *Clin Exp Dent Res* **2021,** *7* (5), 845-851.

**Supplementary Table S4. Quality assessment of the included studies by Newcastle–Ottawa scale.**

| Author  (Publication Year) | Newcastle-Ottawa Scale | | | | | | | | | |
| --- | --- | --- | --- | --- | --- | --- | --- | --- | --- | --- |
|  | Selection | | | Comparability | | | Outcome | | | Total |
|  | a | b | c | d | e | f | g | h | i |  |
| Yu 2015 | * | * |  |  | * |  | * | * | * | 6 |
| Batty 2018 | * | * | * | * |  |  | * | * | * | 7 |
| Beck 1996 | * | * |  |  |  | * | * | * | * | 6 |
| Choe 2009 | * | * | * |  | * | * | * | * | * | 8 |
| Destefano 1993 | * | * |  |  | * | * | * | * | * | 7 |
| Dietrich 2008 | * | * | * |  | * |  | * | * | * | 7 |
| Heitmann 2001 | * | * |  |  | * |  | * | * | * | 6 |
| Hung 2004 | * | * |  | * | * |  | * | * | * | 7 |
| Hung 2003 | * | * | * | * | * |  | * | * |  | 7 |
| Jimenez 2009 | * | * |  | * | * | * | * | * |  | 7 |
| Joshipura 2003 | * | * |  |  | * | * | * | * | * | 7 |
| Joshipura 1996 | * | * | * |  | * |  | * | * | * | 7 |
| LaMonte 2017 | * | * | * | * | * | * | * | * | * | 9 |
| Noguchi 2014 | * | * | * | * | * |  | * |  |  | 6 |
| Rivas-Tumanyan 2012 | * | * | * | * | * |  | * | * |  | 7 |
| Senba 2008 | * | * |  |  |  |  | * | * | * | 5 |
| Andriankaja 2007 | * | * | * | * | * | * | * |  |  | 7 |
| Tuominen 2003 | * | * | * |  | * |  | * | * | * | 7 |
| Morrison 1999 | * | * |  |  | * | * |  | * | * | 6 |
| Beck 2005 | * | * | * |  | * | * |  | * | * | 7 |
| Chen 2016 | * | * |  | * | * | * | * | * | * | 8 |
| Hansen 2016 | * | * | * |  | * | * | * | * | * | 8 |
| Joshy 2016 | * | * | * |  | * | * | * | * | * | 8 |
| Howell 2001 | * | * |  |  | * | * |  | * | * | 6 |
| Lee 2013 | * | * | * | * |  |  | * | * | * | 7 |
| Ahn 2015 | * | * | * |  |  | * | * | * | * | 7 |

1. Representativeness of the exposed cohort.
2. Selection of the non-exposed cohort.
3. Ascertainment of exposure.
4. Demonstration that outcome of interest was not present at start of study.
5. Comparability of cohorts on the basis of the design or analysis (adjusted for age).
6. Comparability of cohorts on the basis of the design or analysis (adjusted for any other factor).
7. Assessment of outcome.
8. Was follow-up long enough for outcomes to occur.
9. Adequacy of follow-up of cohorts.

**Supplementary Table S5. GRADE evidence profile**

| **Certainty assessment** | | | | | | | **№ of patients** | | **Effect** | | **Certainty** | **Importance** |
| --- | --- | --- | --- | --- | --- | --- | --- | --- | --- | --- | --- | --- |
| **№ of studies** | **Study design** | **Risk of bias** | **Inconsistency** | **Indirectness** | **Imprecision** | **Other considerations** | **periodontal disease patients** | **non-periodontal disease patients** | **Relative (95% CI)** | **Absolute (95% CI)** |  |  |
| **Risk of CVD (follow-up: range 2 to 34 years; assessed with: ICD 9 or ICD 10)** | | | | | | | | | | | | |
| 20 | observational studies | not serious | serious | serious | not serious | none | 492519/2773424 (17.8%) | 422370/2773424 (15.2%) | **OR 1.25** (1.13 to 1.38) | **31 more per 1,000** (from 16 more to 46 more) | ⨁◯◯◯ Very low | CRITICAL |

**CI: confidence interval; OR: odds ratio**

**Supplementary Figure S1. Forest plot of the prevalence of cardiovascular disease in patients with periodontal disease.**

**(A: CHD; B: Stroke; C: HP; D: HF).**

**
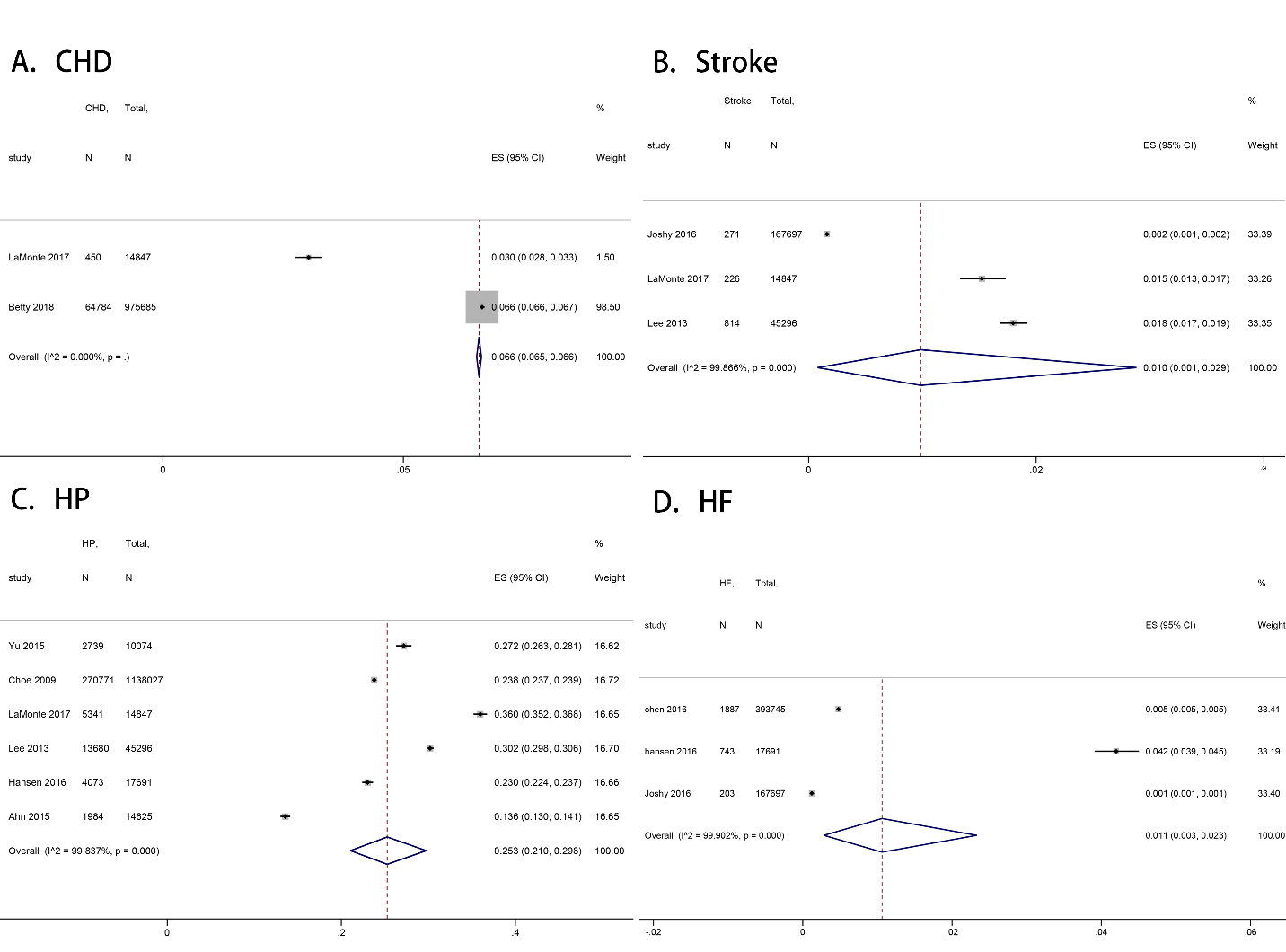
**

# CHD: coronary-heart-disease; HP: hypertension; HF: heart failure

**Supplementary** **Figure S2. Publication bias detected by funnel plot, Egger’s test and Begg’s test for the sex-specific association between periodontal disease and CVD.**

**
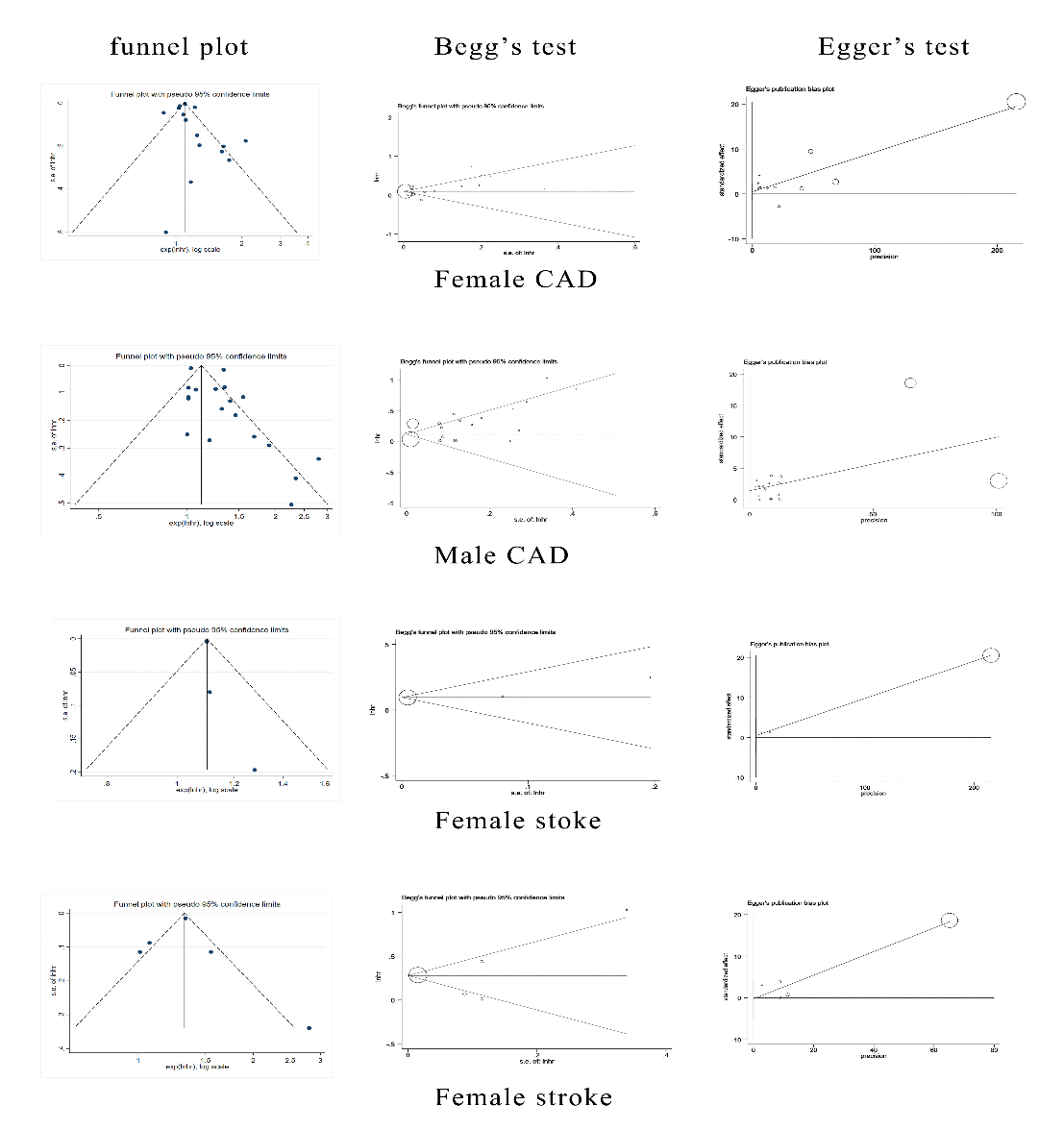
**

**Supplementary** **Figure S3. Sensitivity analysis of the association between periodontal disease and CVD.**

**A: Female periodontal disease and CVD; B: Male periodontal disease and CVD**

**
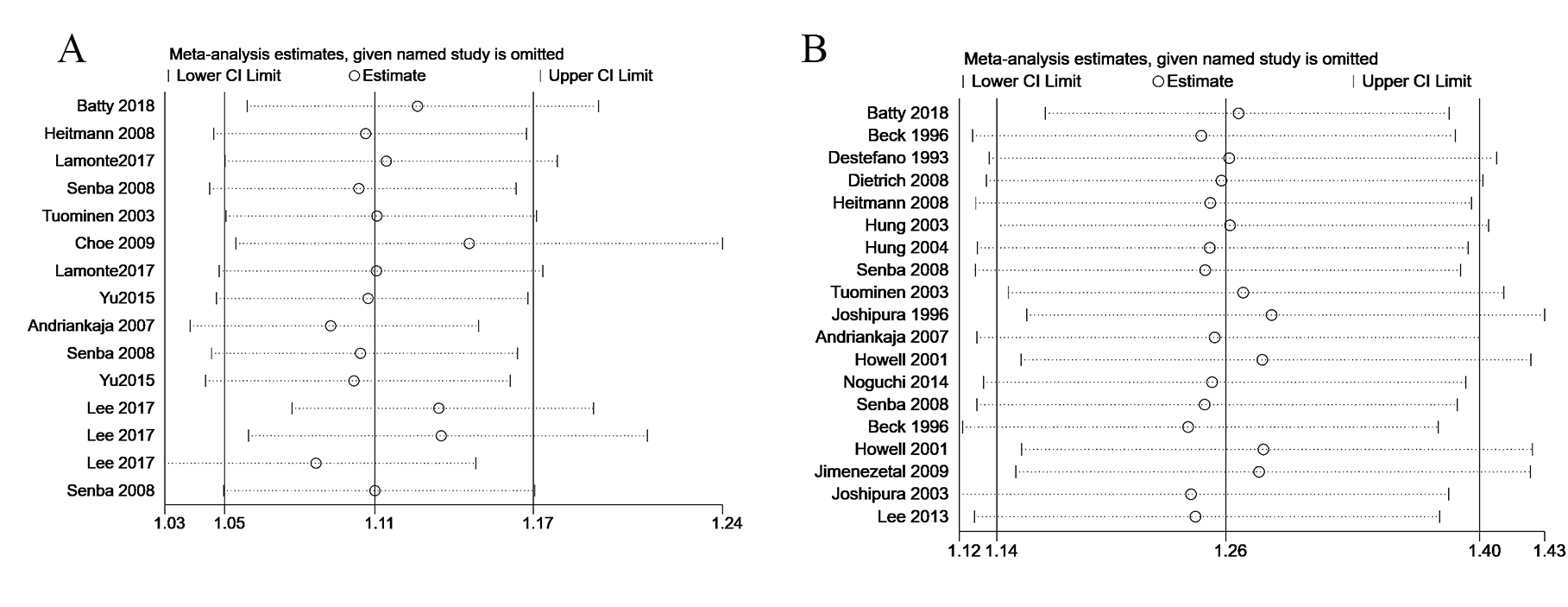
**
